# Musical Episodic Modulation of Human Functioning: Anticipation Mechanisms, Personal Sense, and Emotional Recall

**DOI:** 10.1007/s12124-026-10033-0

**Published:** 2026-10-01

**Authors:** Rodrigo Drumond Vieira

**Affiliations:** Fluminense Federal University (Society, Education and Knowledge), Niterói, Rio de Janeiro, Brazil

**Keywords:** Activity theory, Anticipation mechanisms, Floating intentionality, Musical episodic modulation, Phenomenology, Psychology of music

## Abstract

This article develops a theoretical and exploratory account of musical episodic modulation of human functioning, with special attention to emotional recall. It responds to a recurrent limitation in the psychology of music: although music’s effects on attention, emotion, memory, motivation, and performance have been widely documented, these domains are often examined separately rather than as mutually organized dimensions of lived episodes. The article proposes that music may function as a *modulating agent* of human functioning when its temporal, affective, and semantically open structure becomes *coupled* with ongoing activity through *anticipation mechanisms.* Drawing on activity theory, phenomenology of temporal consciousness, floating intentionality, entrainment, and appraisal theory, the paper conceptualizes *anticipation mechanisms* as the dynamic relations among anticipators, the anticipated, and horizonality. These mechanisms explain how musical signals may organize salience, attention, affect, readiness, and possible next actions over time. The argument is illustrated through a phenomenologically oriented self-study in which the same song contributed to opposed emotional episodes during two recall activities: hopelessness, yearning, and sadness in relation to the memory of a deceased father, and excitement, hope, and joy in relation to an anticipated romantic encounter. The contrast suggests that musical valence does not determine emotional outcome by itself. Rather, emotions emerge through projection-appraisal loops involving objects of thought, personal sense, belief systems, previous experience, and the unfolding music. The article concludes by outlining limitations, future research directions, and distinguishing the more specific phenomenon analyzed from the broader account of musical modulation of human functioning.

## Introduction

People often listen to music while acting, and the psychology and the sociology of music have described music’s everyday uses (DeNora, 2000; North et al., 2004; Schäfer et al., 2013; Sloboda & O’Neill, 2001). Music can improve several psychological processes, including sustaining attention, enhancing emotional and cognitive functioning, regulating arousal, and positively affecting work and physical activity (Large & Jones, 1999; Kiss & Linnell, 2021; Lesiuk, 2005; Särkämö et al., 2008; Terry et al., 2020). Cognitive neuroscience has shown that preferred music strongly influences reward and motivation (Salimpoor et al., 2011; Zatorre & Salimpoor, 2013). Research has also shown that music can positively influence perception, creativity, concentration, stress management, and alter memory processes during recollection (Huang & Shih, 2011; Knight & Rickard, 2001; Lesiuk, 2005; Ren et al., 2024). Music may be used as a resource in clinical, therapeutic, and everyday self-regulatory contexts (DeNora, 2000; Gebhardt et al., 2014; See et al., 2023). Listening to music has also been associated with correlated neural synchronization (Weineck et al., 2022).

These research lines are indispensable. Yet a broader theoretical issue remains insufficiently articulated: existing research has often examined music’s effects on specific processes, while less attention has been given to how music may organize cognition, emotion, embodiment, and action together within temporally unfolding episodes of human functioning (Vieira, 2023, 2027). The present article advances a theoretical answer to this problem. I propose that music may function as a *modulating agent* of human functioning. By this expression, I mean that music can maintain or alter salience, anticipation, readiness, affective tone, directionality, and possible next actions within an unfolding episode. Music does not become modulating simply because it is physically present. It becomes modulating when it enters the horizon of an activity and is functionally coupled with what a person is doing or preparing to do. In such cases, music may help organize the temporal and structural course of experience itself.

The concept developed here is musical episodic modulation of human functioning. I first introduced the label “musical episodic modulation” in a Portuguese study (Vieira, 2023). It refers to the process by which temporally structured musical signals interact with *anticipation mechanisms* and thereby participate in organizing cognition, emotion, embodiment, and conduct into coordinated episodes. The term episodic is crucial. It indicates that the relevant unit is not an isolated stimulus-response event, but a bounded episode with a beginning, development, transformation, and closure. A musical piece, or a meaningful musical phase, may become integrated with the episode, supporting alignment between the temporal unfolding of music and the temporal unfolding of experience.

This theoretical problem also requires an inquiry mode that attends to lived temporal organization. For this reason, the present article includes a phenomenologically oriented self-study of two recall episodes in which I listened to the same song while focusing on different objects of thought. The self-study is not a stand-alone empirical demonstration or a basis for statistical generalization. Rather, it serves as an exploratory, descriptive field in which the theory becomes analytically visible. Its purpose is to examine how musical temporality, remembered objects, affective tone, personal sense, and appraisals may become organized together within concrete episodes of experience. Personal sense is understood in Leontiev’s (1978) conceptualization, which will later be distinguished from objective meaning.

The phenomenological orientation of the self-study is important because musical episodic modulation is not directly reducible to isolated variables such as stimulus properties, reported emotion, or behavioral output. It concerns the temporal organization of lived functioning: how a person is directed toward an object, how that object becomes affectively and personally significant, how the musical content and structure enter the horizon of experience, and how the episode develops through changing anticipations and appraisals. In the self-study, the same song was deliberately held constant, while the remembered object and its personal sense changed. This contrast allowed the analysis to explore how one and the same musical piece participated in the emergence of opposed emotional episodes.

This article builds on and substantially expands an earlier account of musical episodic modulation of emotions during recall activities (Vieira, 2023), developing a broader theoretical framework that extends the original analysis in several ways. First, it shifts the focus from emotion alone to human functioning as an integrated temporal organization of psychological processes. Second, it develops anticipation mechanisms as the central explanatory concept. Third, it clarifies how music becomes a modulating agent in the self-study through temporal coupling, entrainment (Repp & Su, 2013), floating intentionality (Cross, 2005), personal sense (Leontiev, 1978), and appraisal (Scherer et al., 2001). Fourth, it frames the phenomenologically oriented self-study as an exploratory illustration of a broader theory of how music may enter, sustain, redirect, or transform episodes of human functioning.

For these aims, this study addresses three interrelated questions:

1. Under what conditions does music become a modulating agent rather than remaining a mere background stimulus?
2. What psychological architecture explains how musical signals become coupled with objects of thought, personal senses, and appraisals?
3. How can the same musical piece participate in the production of opposed emotional episodes during recall?

## From Effects of Music to Musical Episodic Modulation

The psychology of music has generated extensive evidence that music is psychologically consequential (DeNora, 2000; Gebhardt et al., 2014; Huang & Shih, 2011; Huron, 2006; Knight & Rickard, 2001; Koelsch, 2012, 2014; Large & Jones, 1999; Leman, 2007; Lesiuk, 2005; London, 2004; Patel, 2008; Ren et al., 2024; Repp & Su, 2013; Salimpoor et al., 2011; See et al., 2023; Zatorre & Salimpoor, 2013). Yet a theoretical gap remains. Much research asks what music does to specific processes: Does it improve mood? Does it increase arousal? Does it reduce stress? Does it facilitate performance? Does it alter emotional memory? These are important questions, but they do not fully address how music participates in organizing an entire episode of functioning. Human experience is not a set of psychological modules activated one after another. It is an unfolding organization in which several processes operate together in time.

The concept of musical episodic modulation is intended to name and explain this more integrated phenomenon. It does not deny the importance of discrete effects. Rather, it treats them as components of a larger temporal organization. When a student listens to music and writes at the computer, the music may sustain attention, regulate arousal, cue pace, evoke confidence, and mark transitions among work phases. While two people clean a room to a fast, familiar song, musical rhythm may support coordinated motor operations, and musical phases may help organize preparation, execution, and closure. When a person remembers someone while listening to a song, the music may scaffold the temporal unfolding of recall, intensify personal sense, and participate in emotional appraisal. Evidence for these component processes includes context-sensitive effects of background music on attention and task performance (Kiss & Linnell, 2021), rhythm mechanisms in interpersonal coordination (Keller et al., 2014), and music-related changes in memory and emotion (Ren et al., 2024).

Here, modulation refers to a process of maintaining, intensifying, weakening, redirecting, or reorganizing human functioning through coupling. This operational definition of modulation extends established uses of regulation in emotion research, where processes maintain or modify affective states (Gross, 2015), and in research on music influence on emotions, where effects vary with mechanism, listener, and context (Carlson et al., 2015; Juslin & Västfjäll, 2008). From this perspective, modulation does not mean simple causation. A musical stimulus does not mechanically produce a predetermined response. Rather, music enters into a dynamic relationship with the person’s functioning framed by what Husserl (1964) termed a horizon of possibilities. For example, depending on this relationship, the same song may help one person concentrate and distract another. It may evoke sadness in one context and joy in another. This variability is not an obstacle to theory – it is one of the phenomena the theory must explain.

Musical modulation can be loosely or tightly coupled with processes of human functioning. For the latter circumstance, it can become episodic. The term episodic indicates that modulation occurs within bounded temporal organizations. An episode may be short, as in a six-minute recall activity, or longer, as in a period of exercising or film spectatorship. Within episodic modulation, music is an agent that can organize human functioning in time. This use of the term “episodic” relates to van Dijk’s (1982) discursive definition of episode and resonates with event-cognition research, which treats ongoing experience as segmented into internally structured events across multiple timescales and locates event boundaries as meaningful to perception and memory (Zacks, i^2020^). In this sense, musical episodic modulation may facilitate event representation because musical markers of beginning and ending are coupled with the corresponding events in human functioning, thereby reinforcing these boundaries in perception and memory.

It is essential to distinguish musical episodic modulation from a generic claim that music influences people. The present framework proposes that music becomes a modulating agent when its temporal structure is functionally and tightly coupled with the temporal organization of human functioning. It may involve explicit attention to music, peripheral attunement, or a fluctuating relation between focal and background awareness. What matters is that music functions as a constitutive condition for the episode’s unfolding. Research on rhythmic interpersonal coordination (Keller et al., 2014), sustained attention (Kiss & Linnell, 2021), and music-evoked memory (Belfi et al., 2016) supports the broader premise that musical structure can become integrated with motor, attentional, and autobiographical processes, although the form and strength of integration vary across tasks and listeners.

Meta-analytic and behavioral evidence indicates that background music can help, hinder, or leave performance unchanged depending on task demands and listener characteristics (Kämpfe et al., 2011; Kiss & Linnell, 2021). Clinical and neurobehavioral research also shows that some music-listening strategies can be linked to maladaptive emotion regulation rather than improvement (Carlson et al., 2015). Therefore, the central claim is not that music always improves functioning. A slow, melancholic song may support reflective writing for one person and evoke debilitating sadness for another. A fast and bright song may support routine motor operations but impair complex analytical thought. The key theoretical task is to identify the conditions under which music acquires a modulating force and the mechanisms by which this force is integrated into human activity.

## Human Functioning as Temporally Organized Activity

I proposed a theory of musical episodic modulation of human functioning (Vieira, 2027), and it requires an account of what is being modulated. Human functioning is understood as an integrated organization of psychological processes within situated activity. This understanding draws first on Leontiev’s account of the macrostructure of human activity within his original approach to activity theory. According to him, this macrostructure involves three non-additive “units”: activity, action, and operation (Leontiev, 1978, 1981). Activity is rooted in a need, whether biological or sociocultural, and elicited by a material or ideal object, its motive. Action is directed toward a prior or emergent conscious goal. Operations are the methods through which actions are accomplished under specific material and symbolic conditions.

Leontiev’s seminal contribution was establishing a systematic activity theory that conceptualized human activity as an object-oriented, culturally mediated, and socially organized system (Wertsch, 1981). Subsequent scholarship conducted under the interdisciplinary umbrella of *cultural-historical activity theory* has preserved Leontiev’s foundational theoretical architecture while extending its application, particularly to work and educational settings and to human–computer interaction (Engeström, 1999; Nardi, 1996; Vieira & Kelly, 2014). Rather than fundamentally reformulating Leontiev’s account, this scholarship has selectively foregrounded and further elaborated dimensions already present in, or consistent with, his theory, including the collective organization, cultural mediation, historicity, and transformation of human activity (Engeström, 2001; Roth & Lee, 2007; Sannino & Engeström, 2018).

Engeström’s activity-system diagram (Engeström, 1999, p. 31), for example, made the relations among mediating artifacts, rules, community, and the division of labor more visually explicit and analytically accessible. It should therefore be understood as an explicit schematization of Leontiev’s activity theory. Although Leontiev acknowledged the roles of signs and language, his theory is not semiotic in the strict sense because object-oriented practical activity—not semiotic mediation—remains its primary explanatory principle (Glassman, 1996; Rückriem, 2010). Bronckart (1999) complemented Leontiev’s theory by conceptualizing language as a semiotic form of human activity realized through texts and discourse. Vieira and Kelly (2014), and subsequently Melo and Vieira (2025), integrated this perspective into a method for discourse analysis that connects Leontiev’s activity–action–operation structure with Gumperz’s (1982) sociolinguistic account of contextualization in interaction and Adam’s (2008) text-linguistic model of the sequential organization of texts.

Although A. N. Leontiev (1978, 1981) referred to activity, action, and operation as the “units” that form the macrostructure of human activity, subsequent scholarship, particularly Wertsch (1981) and Engeström (1987, 1999), popularized the description as “levels” of analysis. Wertsch did not simply replace one term with the other, since functionally defined units can be identified at different analytical levels. Nevertheless, the widespread use of “levels” may invite an overly vertical interpretation in which activity is positioned above action and action above operation, thereby suggesting a fixed hierarchy in which each level unidirectionally determines the one below it (A. A. Leontiev, 2006). Such an interpretation risks obscuring the dynamic and non-additive character of Leontiev’s formulation. The three units differ primarily in their functional determinants: an activity is oriented by its object-related motive, an action by its consciously represented goal, and an operation by the concrete conditions and means through which that goal is attained. They consequently address complementary analytical questions: motive explains why an activity exists; actions indicate what is being done and toward which conscious purposes; and operations reveal how and under what conditions those actions are accomplished (Adeeb & Vieira, 2024). Their relations are structurally ordered, but they do not constitute a hierarchy of discrete or additive elements: activity exists through actions, actions are realized through operations, and the same process may move from one unit to another as its motive, goal, or conditions change (Leontiev, 1978). The present article therefore retains Leontiev’s original term “units” to preserve their functional differentiation, mutual constitution, and dynamic transformation, while avoiding the reification of activity, action, and operation as fixed hierarchical strata.

This account is useful because it prevents the analysis from reducing music’s influence to isolated mental states. If a person listens to music while remembering a deceased loved one, the relevant process is not simply an internal emotion produced by an external stimulus. It is an activity of remembering, organized around a thought object with personal sense. If one listens to music while assembling a family album, the relevant processes include motives, goals, appraisals, decisions, memories, values, bodily operations, and conditions of production. Music may modulate any of these dimensions within the organization of the activity.

Leontiev’s (1978) distinction between objective meaning and personal sense is also central. Objective meaning refers to socially shared meanings stabilized in language and culture. Personal sense refers to what an object, event, or activity means for a person in relation to motives, needs, values, and lived history. This distinction between socially available meaning and lived personal sense also resonates with semiotic approaches to sense-making, which hold that emotion contributes to the psychological value by which signs, situations, and objects become meaningful for the person in context (Salvatore & Venuleo, 2008). A song may have public meanings attached to genre, style, cultural circulation, or lyrical themes. However, its modulating force in a given episode depends on how it becomes related to personal sense and to the appraisal processes through which that personal sense is evaluated in context. A melody culturally associated with longing may, for one person in a particular episode, give experiential form to the irreversible absence of a father. In a different episode, the same musical material may orient consciousness toward the anticipation of a possible romantic encounter.

The second theoretical resource is Husserl’s (1964) analysis of temporal consciousness. According to him, experience unfolds through the relation among retention, primal impression, and protention. Retention is the just-past as still held within present consciousness. Protention is the implicit orientation toward what is about to occur. Primal impression is the living present in which experience is given. These moments are not separate compartments. They form the temporal flow of experience. This means that the present is always temporally thick. What has just happened and what is expected to happen are already active in the organization of current experience (Husserl, 1964; Zahavi, 2003). Retention is not recollection of a completed past event, and protention is not a deliberate prediction. Both are pre-reflective intentional constitutive structures of the living present (de Warren, 2009; Husserl, 1964). This distinction matters here: anticipation mechanisms are grounded in Husserl’s conceptualization of internal time consciousness, which concerns a basic temporal directedness that makes continuity, surprise, and fulfillment possible.

This phenomenological insight is crucial for understanding music. Listening to music is intrinsically temporal. A note or phrase is heard in relation to what has just sounded and what is anticipated to come. The listener does not experience each musical signal as an isolated acoustic event. Musical meaning and affect arise through temporal relations. Tension, release, expectation, surprise, continuity, and closure all depend on the interplay of retention and anticipation. Music is therefore exceptionally well suited to modulate experience because it enters, in an exemplary way, the temporal structure of consciousness itself, enabling temporal organization (Huron, 2006; Large & Jones, 1999), entrainment potential (Repp & Su, 2013), and semantic openness (Cross, 2005).

Husserl’s (1964) notion of horizonality further clarifies this process. Every object of consciousness is given within a horizon of possibilities, expectations, backgrounds, and implicit meanings. The object is never exhausted by what appears at a single moment. It is surrounded by what may become relevant, what can be anticipated, what is remembered, and what remains latent. In musical episodic modulation, horizonality is shaped by the person’s current activity, the musical unfolding, cultural knowledge, personal history, bodily state, mood, and the object of thought. These dimensions enrich and constrain what can be felt, remembered, anticipated, and enacted. Important to note that scholarship cautions against treating Husserl’s tripartite structure of retention-primal impression-protention as a ready-made cognitive mechanism. Attempts to translate it directly into predictive-processing models remain contested because phenomenological description operates at the level of lived experience, whereas computational accounts operate at a subpersonal explanatory level (Bogotá, 2025; Grush, 2006).

Combining Leontiev and Husserl allows us to conceptualize human functioning as activity and consciousness unfolding temporally. Leontiev’s (1978) account of activity supplies motives, goals, operations, and personal sense. Husserl’s (1964) analysis of temporal consciousness conceptualizes it as a tied process of retention, protention, present experience, and horizonality. Music can modulate human functioning because it can be coupled with both dimensions: it may participate in the activity’s organization and in the temporal flow through which activity and consciousness are lived in bounded episodes. Both frameworks are therefore used selectively. Developments in cultural-historical activity theory have extended Leontiev’s account to emphasize activity systems and their historically developing contradictions (Engeström, 2001; Sannino & Engeström, 2018). Husserl’s phenomenology of time-consciousness, as examined by de Warren (2009), offers resources for understanding the temporal structure of experience and its constitutive role in subjectivity. Together, these frameworks contribute to the conceptual vocabulary used here to develop a theory of musical episodic modulation of human functioning.

## Anticipation Mechanisms and Coupling

The present framework’s core explanatory resource is the concept of anticipation mechanisms. These mechanisms refer to the dynamic organization through which what has just been accomplished and retained functions as an anticipator of what may come next within a structured horizon of possibilities. Anticipation mechanisms involve three interrelated components: anticipators, the anticipated, and horizonality.

Anticipators are retained elements that orient ongoing functioning. They may include musical signals just heard, words just thought or spoken, motor operations just accomplished, emotional tones already established, remembered scenes, or appraisals. The anticipated refers to what is projected as possible, expected, imminent, or prepared. Horizonality refers to the broader field of possibilities and constraints in which anticipators and anticipated take shape. This field includes music’s temporal structure. In future-oriented episodes, anticipation mechanisms may also involve imagination, understood not as detached fantasy but as a culturally mediated process that relates past experience, present concerns, and possible future courses of action and experientially elaborates them (Pelaprat & Cole, 2011).

In musical experience, anticipators may include a melodic phrase, a harmonic progression, a lyrical theme, or a remembered phase of a known song. The anticipated may be the return of a chorus, the arrival of a vocal climax, the beginning of a solo, a change of tempo, or the expected closure of the song. Such a conceptualization aligns with research on musical expectation and predictive coding, which supports the premise that familiar temporal patterns generate hierarchically organized expectations whose confirmation or violation shapes attention, action, and affect (Huron, 2006; Koelsch et al., 2019; Vuust et al., 2022). In non-musical activity, anticipators may be procedures just completed, thoughts just formulated, or emotions already activated. Anticipators may potentially be linked to the residual activation or inhibition associated with a recently completed action (Hommel, 2004; Mayr & Keele, 2000), to newly formulated representations maintained in working memory (Soto et al., 2008), or to already activated affective states that influence subsequent choice (Knutson & Greer, 2008). Musical episodic modulation occurs when musical anticipators and activity-related anticipators become functionally coupled.

Cheung et al. (2023) provided a useful empirical differentiation within musical anticipation. Comparing computational models with listeners’ ratings of chord surprise and pleasantness, they found independent contributions from sensory expectations based on acoustic information and cognitive expectations based on learned musical structure. Musical anticipation should therefore not be treated as a single undifferentiated process. In the present framework, these are candidate sources of orientation toward musical continuations; the further proposal is that such orientations may become coupled with personally significant possibilities in activity. Expecting a harmonic continuation and anticipating reunion with another person remain distinct processes whose functional relation requires investigation. The chord-rating findings support this differentiation of musical expectancy, but do not establish autobiographical projection, phenomenological horizonality, or episodic coupling.

The present account also extends entrainment without reducing musical modulation to it. Entrainment refers to the process by which interacting rhythmic processes adjust their timing to establish a stable temporal relationship. In music, this can involve movements synchronizing with an external rhythm (Repp & Su, 2013) or attentional rhythms aligning with the music’s temporal regularities (Large & Jones, 1999). Entrainment provides a temporal basis for musical coupling. However, coupling is broader than synchronization. It refers to the functional integration of musical signals and psychological processes within the same unfolding episode. A person may not visibly move to music and yet may have attention, affect, recall, or decision-making modulated by musical structure. A person may or may not be aware of coupling in musical modulation. Empirical and theoretical work on the influence of music on humans distinguishes strict phase synchrony from broader period entrainment and action-perception coupling and shows that rhythmic coordination can involve anticipatory adjustment across motor, perceptual, and interpersonal levels (Keller et al., 2014; Novembre & Keller, 2014; Thaut et al., 2015). These findings suggest interpreting musical episodic modulation as extended coupling processes in time.

Predictive coding and dynamical-systems approaches offer complementary, but not interchangeable, resources for understanding coupling and anticipation mechanisms at the subpersonal level. Predictive processing conceptualizes perception and action as involving hierarchically organized expectations, precision-weighted prediction errors, and the updating of internal models or actions in response to incoming signals (Koelsch et al., 2019; Palmer & Demos, 2022; Vuust & Witek, 2014). Dynamical-systems approaches emphasize coordination among interacting processes that evolve over time, including phase relations, entrainment, anticipatory synchronization, and transitions between coordination states (Roman et al., 2019; Stepp & Turvey, 2010). These approaches therefore help explain how musical events become expected, why deviations acquire functional significance, and how musical timing may become progressively coordinated with bodily and cognitive activity. In music specifically, contemporary predictive-coding models relate enculturated priors, hierarchical prediction, prediction error, action, and affect (Vuust et al., 2022), drawing on the broader predictive-processing framework in which perception and action are organized through hierarchical prediction and prediction-error minimization (Clark, 2013).

The theory of musical episodic modulation differs from predictive-coding and dynamical-systems approaches primarily in its explanatory focus: the situated organization of a bounded episode in which music becomes functionally integrated with cognition, emotion, embodiment, and action. Predictive coding explains anticipation through hierarchically organized predictions and prediction errors, whereas dynamical-systems accounts explain anticipatory coordination through coupling among evolving processes, without necessarily requiring internal representations of future events (Palmer & Demos, 2022). These resources illuminate how anticipation and coordination occur, but their general principles do not by themselves specify how musical unfolding becomes consequential for a particular object of thought, personal sense, or course of activity. The concept of *anticipation mechanisms* proposes this further specification through the relations among *anticipators*, *the anticipated*, and *horizonality*: retained musical signals, thoughts, appraisals, and accomplished operations jointly shape what becomes possible, salient, or actionable next within a horizon of possibilities structured by motives, goals, personal history, beliefs, and current conditions. Its proposed contribution is therefore to articulate how musical expectations become coupled with personally significant possibilities, such that anticipating a musical intensification or closure may participate in the elaboration of an appraisal, the intensification of an emotion, the redirection of an action, or the completion of recall. In the two self-study episodes, *the same expected musical continuation* entered different horizons of loss and romantic possibility. In this sense, anticipation organized the unfolding projection–appraisal process without independently determining its emotional valence. This formulation of anticipation mechanisms offers a *relational account* of how *temporal coordination* acquires intentional and practical significance through the interplay of objects of thought, personal sense, expectation, musical structure and timeframe, appraisals, emotions, past experiences, and beliefs within a bounded episode, while leaving open the empirical question of whether and how this complex relational organization may be investigated computationally, neurally, and dynamically.

Music becomes a modulating agent when it alters the immediate conditions of what can happen next in consciousness and activity. A musical signal just heard becomes part of the conditions for the next thought, feeling, gesture, decision, or operation, and these modify how music is heard. In this sense, music participates in the ongoing constitution of the episode by integrating the temporal architecture of functioning and entering a person’s lived horizon of personal functional significance and anticipation. In short, this phenomenon is the musical *coupling* with human functioning, whereby it can become a modulating agent. This point is essential because music does not modulate merely by its physical presence. A musical track may go unnoticed, remain peripheral, or be irrelevant. In such a case, it is sound in the environment, but not yet a modulating agent. Thus, musical modulation depends not only on acoustic structure but also on the relations between musical structure and its functional significance in the moment. Familiar music may contribute to modulation by engaging emotional and reward-related processes (Pereira et al., 2011; van den Bosch et al., 2013). Familiarity with a piece can also support the anticipation of upcoming musical events through expectations based on prior listening experience (Huron, 2006; van den Bosch et al., 2013). If the person knows that a bright middle section will arrive, a vocal intensification will occur, or a soft ending will close the piece, these anticipated musical events may be coordinated with activity phases (Vieira, 2023). Familiarity is not required, but it facilitates coupling by improving anticipation.

Musical modulation, including episodic modulation, should not be conflated with the mere temporal coincidence between a musical event and a momentary change in thought, action, or emotion. Because both music and human functioning unfold continuously over time, isolated coincidences can readily occur. For example, a listener may decide to send an urgent message to an acquaintance precisely when a musical phrase reaches closure. Such simultaneity does not, by itself, demonstrate that the musical event contributed to the decision; the correspondence may be entirely coincidental. Musical modulation can be inferred only when analysis reveals a temporally structured and functionally coherent coupling through which changes in musical organization systematically shape, constrain, or redirect the unfolding of cognitive, motor, or affective processes. Modulation therefore entails a functional temporal relationship between music and human functioning, rather than mere simultaneity. Episodic modulation is a more specific case in which this coupling constitutes a bounded episode, and the music’s temporal organization becomes functionally coordinated with the relatively stable or dynamically evolving demands of an activity or action.

## Floating Intentionality, Projection, Appraisal, and Emotion

Although music can be culturally meaningful, expressive, and associated with actions, places, identities, emotions, or narratives, it generally specifies much less determinate propositional reference than language (Slevc & Patel, 2011). Cross (2005) conceptualized this open-ended “aboutness” as *floating intentionality*. According to him, music “can be thought of as gathering meaning from the contexts within which it happens and in turn contributing meaning to those contexts (2005, p. 30)”.

Floating intentionality is central to explaining the variability of emotional responses to music. Because music’s aboutness remains partly underdetermined, the same piece may afford different meanings to different listeners, or even to the same listener across different episodes (Cross, 2005). This interpretive openness does not render musical meaning arbitrary; rather, it allows meaning to be shaped in context through musical features and listeners’ experiences (Clarke, 2005; DeNora, 2000). A song may accordingly afford several emotional possibilities. Which of these possibilities becomes salient depends on both the musical features and the situational mechanisms through which the listener engages with them (Juslin & Västfjäll, 2008).

The concept of projection helps describe how this specification occurs. In recall activities, the remembered object evokes associated experiences, values, and beliefs (Vieira, 2023, 2027). In musical listening, listeners may project these contents onto the musical experience (Fiske, 1996). Projection does not mean that the listener simply adds private meaning to a neutral stimulus. Rather, it describes a two-way process: the projected contents give the music personal significance, and the music gives them temporal, affective, and expressive shape. Here, projection is used in a restricted relational sense specific to the present account, not as the psychoanalytic defense mechanism through which internally unacceptable contents are attributed to an external source (Freud, 1962). It denotes the contextual specification of musically afforded meanings by autobiographical and activity-related significance. Ecological and sociological approaches likewise describe musical meaning as emerging in transactions among sound, listener, action, and situation (Clarke, 2005; DeNora, 2000), and studies of music-evoked autobiographical memory show how songs can cue memories of specific events from the listener’s life (Belfi et al., 2016; Janata, 2009).

The Constructivistly-Organized Dimensional-Appraisal (CODA) model (Lennie & Eerola, 2022) provides a music-specific connection between appraisal and the significance of an episode. This model emphasizes that emotional responses to music depend on how listeners evaluate what they experience in relation to their goals and concerns, a process termed *appraisal*. Because these goals and concerns differ across individuals and situations, the same music may be appraised differently and contribute to different emotional trajectories. In the present framework, this variability is further examined through personal sense: the recalled person’s significance to the listener, shaped by motives and lived history, informs how the content of recall is appraised in relation to the listener’s goals and concerns during the musical episode.

I will introduce an example from my music-modulated recall episodes to clarify the role of projection, personal sense, and appraisal in constructing emotion. The remembered father is not simply a category such as “father”. Rather, he is a unique person whose meaning is shaped by love, biography, absence, and values. This personal sense is the experiential material projected onto music. Appraisal theory (Lazarus, 1991; Scherer et al., 2001) then clarifies how emotion is constructed. Appraisal approaches argue that emotions arise from appraisals (i.e., evaluations) of events, objects, or situations in relation to concerns, goals, values, and coping possibilities. In musical episodic modulation, the object of appraisal is not music alone. The appraised object is the personal sense projected onto music, elaborated through the relation among the object of thought, the person’s history, the current activity, and the unfolding music. Personal sense and appraisal are distinguished here by their functions within an episode. Personal sense concerns what an object means for the person in relation to motives and lived history. It draws on previous experiences, including earlier appraisals. Appraisal concerns the ongoing evaluation of that personally significant object in relation to current concerns and possibilities, such as whether reunion is possible or loss is irreversible. Personal sense thus specifies the object’s motivational and biographical significance, whereas appraisal specifies how its current or anticipated circumstances bear on the person’s concerns. Their relationship is recursive: the projected personal sense onto music orients appraisal, while appraisal may transform personal sense as the episode unfolds. The same song may participate in sadness when the appraised personal sense concerns irreversible loss and no possibility of reunion, and joy when the appraised personal sense concerns a possible encounter.

Therefore, musical valence does not mechanically determine emotional valence. The emotional outcome depends on the projection-appraisal loop. Musical features guide temporal attention and affective orientation, but the emergent emotion depends on how the person appraises the personally significant object within the musical horizon. In alignment with this assumption, reviews of music-and-emotion research distinguish emotions perceived in music from emotions felt by listeners and highlight the substantial roles of context, familiarity, goals, and individual differences. Sad-sounding music, for example, may be experienced with pleasure or mixed affect rather than straightforward sadness (Eerola & Vuoskoski, 2013; Juslin & Västfjäll, 2008).

The phrase *projection-appraisal loop* names the following dynamic. First, an object of thought becomes focal within an activity. Second, this object evokes a personal sense shaped by previous experiences, beliefs, values, and motives. Third, the personal sense is projected onto music whose floating intentionality allows coupling and contextual specification. Fourth, music structure and its floating intentionality feed forward into the personal sense by shaping its temporal and affective development. Fifth, the evolving personal sense is appraised consciously or unconsciously. Sixth, emotion emerges, is sustained, transformed, or extinguished in coordination with the musical unfolding. Seventh, the emotion itself becomes a new anticipator, altering the next phase of thought, memory, bodily state, and appraisal. This loop should not be understood as a rigid sequence. In lived experience, the components operate together and recursively. Projection may already contain appraisal. Appraisal may redirect projection. Music may change the object of thought. Emotion may alter the horizon of anticipation. Still, the model is useful because it identifies the processes through which music’s open intentionality becomes emotionally specific.

Céspedes-Guevara (2023) distinguished general changes in pleasure–displeasure and activation–deactivation, termed core affect, from specific emotional experiences. Feeling more activated or experiencing discomfort during music listening does not, by itself, establish which emotion the listener is experiencing. Associations and appraisals help relate these affective changes to particular memories, circumstances, and concerns, contributing to the construction of a personally meaningful emotional experience. This distinction helps clarify the recall episodes examined here. Remembering the father or the woman brings different autobiographical contents into the musical experience, while appraising whether reunion is possible helps differentiate irreversible loss from an attainable encounter. In the present framework, projection describes how these personally significant contents shape what the music is experienced as being about, while the music’s expressive qualities and temporal unfolding shape how those contents are experienced. Changes in the listener’s pleasure and activation may thus interact with autobiographical associations and appraisals as music unfolds within the episode.

## Constructs in Musical Episodic Modulation: A Synthesis

Several theoretical constructs and concepts were approached to circumscribe the phenomenon of musical episodic modulation of human functioning. Table 1 synthesizes them and specifies their proposed functional definitions, roles in the theoretical framework, relationships to other constructs, and possible empirical manifestations. Roles distinguish processes, conditions, experiential contents, and outcomes. They do not imply independent or sequential modules. Empirical manifestations are candidate indicators, not validated diagnostic criteria. Recurrence, functional coherence, convergent measures, and, where feasible, experimental perturbation are needed to distinguish modulation from mere temporal coincidence. A thought, appraisal, or emotion may be an outcome at one moment and an anticipator at the next. This role shift is part of the proposed mechanism. Projection, floating intentionality, personal sense elaboration, appraisal, and emotional change specify the analyzed emotional recall pathway, not a universal sequence required of every instance. Table 1 is divided into parts A, B, and C according to thematic organization.

**Table 1:** Proposed functional definitions, roles, relationships, and empirical manifestations

**Construct**	**Functional definition**	**Role in the theoretical framework**	**Relations to other constructs**	**Possible empirical manifestations**
**A. Phenomenon, activity, and context**				
**Musical episodic modulation**	Music contributes to the organization of human functioning within a bounded episode through functional temporal coupling.	Target phenomenon: episode-level organization.	Integrates musical unfolding with anticipation, cognition, emotion, embodiment, and/or action.	Coherent relations between musical signals and phases and the development, transitions, or closure of activity.
**Activity**,** action**,** and operation**	Activity is oriented by an object-related motive; action by a conscious goal; operation by concrete conditions and means.	Functional units of situated activity, not separate psychological states.	Specify why the episode matters, what is being accomplished and for what purposes, and how. Music may affect their relations and execution.	Accounts of the object-related motive sustaining an activity; identifiable actions; and observable procedures through which goals are pursued, including changes in their pace, sequence, or execution as music unfolds.
**Object of thought**	The person, event, possibility, or issue toward which thinking is directed during an episode.	Intentional focus; distinct from its personal sense or appraisal.	May express the activity’s motive in recall, but need not coincide with it in every activity. Evokes personal sense, which may be appraised.	Identifiable referents in recall reports or discourse; shifts between remembered people, scenes, or imagined encounters.
**Musical structure and timeframe**	The unfolding arrangement of rhythm, melody, harmony, dynamics, phrases, and duration, including lyrics where present.	Temporally structured resource; not itself a psychological mechanism.	Provides possible anticipators and expressive conditions. Its relevance depends on its integration with activity.	Musical annotated onsets, intensifications, repetitions, transitions, and endings aligned with measures of the episode.
**Horizonality**	The changing field of possibilities and constraints within which retained elements orient what may occur next.	Contextual and experiential organization within anticipation mechanisms.	Includes motives, goals, beliefs, personal history, bodily state, cultural knowledge, discourse, and musical unfolding; changes as the episode develops.	Reports and contextual evidence of possibilities regarded as available, expected, excluded, or relevant at successive moments.
**Construct**	**Functional definition**	**Role in the model**	**Relations to other constructs**	**Possible empirical manifestations**
**B. Anticipatory organization and coupling**				
**Anticipation mechanisms**	Dynamic relations through which retained elements orient forthcoming possibilities within horizonality.	Proposed relational mechanism at the experiential level.	Connect anticipators with the anticipated; coordinate musical and activity-related processes without independently fixing emotional valence.	Convergent evidence of preparation for transitions, changes of readiness, or prospective orientation tied to musical and activity events.
**Anticipators**	Retained elements that functionally orient subsequent experience or conduct: musical signals, thoughts, appraisals, emotions, or completed operations.	Relational role; not a separate class of stimulus or state.	Become anticipators through their relation to what follows within a horizon.	A completed musical phrase, thought, or operation systematically precedes and orients a subsequent transition; mere precedence is insufficient.
**The anticipated**	What is experienced or enacted as possible, expected, imminent, or prepared, including musical continuations and activity-related possibilities.	Prospective orientation; not necessarily an explicit prediction.	Arises in relation to anticipators and horizonality. May be fulfilled, revised, frustrated, or abandoned as the episode unfolds. When realized, the anticipated event may become an anticipator that orients the next phase of experience or action	Reports of what the person expects or feels prepared to experience or do next at identifiable moments; observable preparation for an upcoming musical or activity transition; and subsequent fulfillment, revision, or abandonment of that possibility.
**Functional temporal coupling**	Functional integration through which the unfolding temporal structure of music contributes to maintaining or reorganizing activity-related processes within an episode. Through this coupling, music functions as a modulating agent of human functioning.	Core relational process linking music to ongoing human functioning.	Connects musical and activity-related anticipators. Musical events shape readiness or appraisal; activity processes change how subsequent music is experienced.	Repeated, contextually interpretable correspondences between musical phases and changes in attention, appraisal, emotion, or action, supported by convergent experiential and behavioral evidence.
**Entrainment**	Temporal alignment of processes, such as movement with rhythm or attention with pulse.	Possible coordination pathway; narrower than functional coupling.	May support coupling but does not, on its own, establish personal sense, appraisal, or organization of a whole episode.	Observable alignment of movement, attentional fluctuations, or physiological rhythms with the timing or periodicity of musical events.
**Feedforward into anticipation**	The process through which an emerging emotion, appraisal, thought, musical signal, or completed action becomes a condition for the next phase.	Recursive relation, not an independent state or final stage.	Links provisional outcomes to anticipators and horizonality; permits redirection of projection, appraisal, and action.	Sequential evidence that a musical event, developing feeling, or evaluation changes subsequent attention, interpretation, readiness, or conduct.
**Episodic boundaries and phases**	The experienced and enacted beginning, development, transitions, and closure of a relatively bounded activity episode.	Temporal organization of the target phenomenon.	Become functionally related to musical phases. Boundaries need not match every musical event or coincide with an entire song.	Event-boundary reports and behavioral transitions that relate coherently to musical development and completion.
**Construct**	**Functional definition**	**Role in the model**	**Relations to other constructs**	**Possible empirical manifestations**
**C. Personal sense and the emotional recall pathway**				
**Personal sense**	What an object means for the person in relation to motives and lived history, as experienced and elaborated in the present.	Motivational and biographical significance; historically formed and currently evolving.	Draws on past experience, including earlier appraisals; orients current appraisal and may be transformed by it. Distinct from shared objective meaning.	Descriptions of a father as loved and irreplaceable or a person as a desired romantic possibility; changes in such significance.
**Floating intentionality**	The relative openness of musical aboutness to contextual specification, within cultural and modulation-specific semantic constraints.	Interpretive affordance, not an appraisal or an emotional outcome.	Permits different personal senses to inform musical experience. Lyrics can constrain the possibilities without fixing one personal referent.	Different autobiographical referents or personal interpretations of the same song, considered alongside its lyrical and expressive constraints.
**Projection**	The relational process through which personal sense specifies musical experience while music gives that sense temporal, affective, and expressive form.	Proposed bidirectional process in the emotional recall pathway.	Links personal sense with music and its interpretive possibilities; interacts recursively with appraisal rather than simply preceding it.	Accounts connecting musical moments with the elaboration of personally significant content; such reports do not independently establish causation.
**Appraisal**	Evaluation of how a personally significant object or situation bears on current concerns, goals, coping, and possibilities.	Evaluative process; may be conscious or nonconscious.	Uses and revises personal sense. Appraisals of irreversible loss or possible reunion differentiate emotional trajectories within musical unfolding.	Changing evaluations of possibility, loss, relevance, or coping; convergent reports and behavioral evidence, without a unique physiological marker.
**Emotion**	An emerging, sustained, or changing affective configuration associated with appraisal	Experiential state and provisional outcome; may subsequently function as an anticipator.	Develops through projection and appraisal under musical conditions; feeds forward into attention, readiness, personal sense, and horizonality.	Time-resolved reports of sadness or joy, crying, and bodily changes.

## Methodological Position: A Phenomenologically Oriented Self-Study

The empirical illustration presented in this article takes the form of a phenomenologically oriented self-study examining my reflective experience of two recall activities undertaken while listening to the same song. It is offered neither as a generalizable experiment nor as a complete phenomenological reduction in the strict Husserlian sense. Rather, it constitutes an exploratory, theory-generating analysis intended to describe and explain how lived experience was organized, based on systematic self-observation, retrospective reflection, and conceptually informed interpretation. Here, the term *first-person* refers to disciplined descriptions produced by the experiencer, without assuming that such descriptions provide transparent or unmediated access to the causal mechanisms underlying the experience (Depraz et al., 2003; Varela & Shear, 1999).

Phenomenological inquiry is appropriate here because the phenomenon under consideration is experiential, temporal, affective, and interpretive. It involves how an episode unfolds from within: how attention moves, how an object of thought becomes salient, how personal sense is projected and appraised, how emotion rises or fades, how music structures the felt duration of experience, and how the person recognizes the episode as beginning, developing, peaking, and ending. Situated within idiographic science and cultural-historical psychology, this first-person study treats the single case as an analytically rich configuration through which psychological processes can be examined in their contextual, temporal, and personal organization (De Luca Picione, 2015; Valsiner, 2017).

This orientation also resonates with Luria’s (1987) notion of “romantic science”, which advocates the deep, multi-sided study of a single case as a pathway to understanding the systemic organization of human functioning. In Valsiner’s (2017) “methodology cycle”, scientific inquiry requires an organic fit between the phenomenon under investigation and the method used to approach it. Since the phenomenon examined here is inherently subjective, temporal, affective, and interpretative, disciplined reflective self-observation is methodologically appropriate for tracking the microgenesis of musical modulation of emotions.

At the same time, I must acknowledge the limitations of self-study. The analysis concerns a single person with a long history of listening to music while working, studying, and reflecting. I have unusually strong personal familiarity with musical modulation, and I am a drummer with long-standing sensitivity to musical timing, rhythm, and transitions. These features do not invalidate the observations, but they define the conditions under which they were produced. The self-study should be read as an analytically rich case of the phenomenon and as a source of hypotheses for future research.

The musical piece used in the self-study was the Brazilian song “Um Dia de Domingo” (in English, “A Sunday”), performed by Gal Costa and Tim Maia, which lasted 6 min and 10 s. I chose it first to remember important people who were absent. I selected my father and the foreign woman to remember soon after and documented the experiences immediately after each episode, on the same day I chose the song. Once both accounts were available, I analyzed each separately. I then compared the resulting analyses to develop comparative interpretations of the two episodes.

I did not anticipate emotional responses before the episodic experiences, as I avoided reflecting on this issue to preserve the spontaneity of my function during the recall episodes. In addition, I did not design the two episodes in advance to produce contrasting emotional experiences, nor was this contrast a research hypothesis. It emerged from the experiences themselves. The original research question that guided the study focused on how emotions unfold during two recall activities while listening to the same song. Before these episodes, remembering my father had also been associated with pride, gratitude, and joy because he had been such a good father to me and the good experiences we had together in the past, even though he was already deceased. Recalling him therefore did not carry the expectation of eliciting sadness or creating an emotional contrast with the second episode. The first approach to this phenomenon was documented by Vieira (2023), which informs the current theoretical interpretation that expands the original analysis to include both a more comprehensive and detailed account of the modulation processes.

The song lyrics evoke the desire to meet, talk with, and be near an absent loved or desired person. Its moderate tempo, warm harmonic ambiance, and emotionally charged melody provide a strong context for recall, longing, and affective projection. I used the same song twice on the same day, once late in the morning and once in the middle of the afternoon. Both occurred while I was alone in my apartment. My previous mood was neutral. The principal difference between the episodes was the objects of thought selected in advance. The episodes were similar in terms of the material conditions of production, but very different in terms of objects of recall, personal sense, and emotions, as described and explained below. To avoid reproducing copyrighted lyrics, I discuss the song only by paraphrasing its general thematic atmosphere.

The first recall activity focused on the memory of my recently deceased father. The second focused on a foreign woman I had met online and wished to meet in person. These objects of thought were associated with very different personal senses, belief systems, horizons, and appraisals. The same musical piece became coupled with two different recall activities and contributed to opposing emotional episodes.

## Self-Study: Two Emotional Episodes with the Same Song

My long-term relationship with music formed the background for the self-study. Since adolescence, I have often listened to music while working on the computer. At first, this involved activities such as designing, diagramming, and programming. Later, during my graduate studies and professional life as a teacher and university professor, I frequently listened to music while writing, reading, reviewing papers, preparing classes, and developing theoretical ideas. Over time, I came to experience music as a powerful resource for disposition, arousal, concentration, attention, creativity, problem-solving, and work performance.

In 2019, I became especially aware that music was not only improving my mood or making work more pleasant. It was modulating the temporal organization of my functioning. During faster, brighter, or more rhythmically intense phases of music, my thinking and writing often accelerated. During slower, denser phases, my pace often slowed, and I became more reflective. Familiar musical signals functioned as cues for possible next operations, and musical transitions often coincided with transitions in thought, decision, or action. This awareness led me to propose that music could participate in the episodic organization of cognition, emotion, and conduct.

The self-study emerged from this background. The temporal course of both episodes appeared to be shaped by patterns of musical salience that guided my temporal attention (Large & Jones, 1999). By directing my attention and perception toward salient musical events, these patterns contributed to the development of the emotional states. Within the song’s timeframe, the *projection of personal senses* and the related appraisal processes followed a trajectory of emergence, elaboration, and closure corresponding to the music’s beginning, development, and ending. This *coupled* temporal organization constituted the episodic modulation of emotions. The following account examines the psychological structure of these processes as I experienced them during self-observations.

My deceased father was the well-defined object of recall in the first episode. The song’s opening coincided with the emergence of a personal sense of him as an immensely loved and irreplaceable person, which I projected onto the music. The song’s subsequent development provided the unfolding musical experience through which this personal sense was progressively elaborated and intensified. Through its projection onto and integration with that experience, the lyrics and melody brought the irreversibility of the loss into conscious appraisal. The atheist belief evoked at that moment also constrained the anticipatory horizon: reunion was excluded as a possibility. From this appraisal arose hopelessness and yearning, whose combination sustained sadness. Crying began by the middle of the song, while the end of the song marked the closure of both my tears and the recall activity.

The second episode involved a different object of recall: a woman I had met online who lived in another country. I had never met her in person, although I had missed opportunities to do so. My memory of her invitation to visit her country formed part of the basis for a personal sense of her as a desired, still-attainable romantic possibility. As the semantically polyvalent music unfolded, this emerging personal sense was progressively elaborated through projection onto, and integration with, the ongoing musical experience. This process contributed to the conscious appraisal of a possible near-future encounter with her. Hope and excitement emerged from this appraisal and together sustained a sense of joy. When the song ended, the recall activity ended as well.

Taken together, the episodes illustrate how music served as a temporally integrative *modulating agent* during the recall activities. Its contribution to the shaping of appraisal processes and emotional states involved *anticipation mechanisms* that organized the temporal unfolding of the projection–appraisal process. As I listened, the retention of previous musical moments, the affective salience of present cues, and the orientation toward expected continuations connected what had already occurred with what was about to emerge. Through this *coupled* relation, anticipation mechanisms coordinated the projection and progressive elaboration of emerging personal senses with the song’s phases, intensifications, and closure. This coordination contributed to the distinct appraisals experienced in the two episodes.

The contrast between the two episodes is the central observation. The same song, heard by the same listener on the same day, in the same physical setting, and from a similarly neutral initial mood, contributed to opposed emotional states. This does not imply that music lacks valence or expressive character. Rather, the contrast suggests that musical valence alone is insufficient to determine the emotional outcome. The emotional states emerged through the relations among musical features, object of thought, personal sense, belief system, horizon of anticipation, and appraisal. Interpreting these episodes requires explicit consideration of the convergence of the song’s lyrics and instrumental expressiveness, whose thematic content and affective qualities were relevant to both objects of recall. The observed modulation must therefore be understood as involving an integrated configuration of lyrical content, musical structure, expressive qualities, and personal associations. The present self-study cannot disentangle the relative contributions of these components. The presence of lyrics also requires a careful specification of *floating intentionality*, since lyrical content provides semantic orientations that shape the song’s interpretive possibilities. Semantic openness does not imply an absence of semantic constraints, and its degree varies across musical stimuli. In this case, the lyrics constrained the interpretive possibilities without fixing a single autobiographical referent or emotional outcome. Their thematic relevance remained compatible with distinct personal senses and appraisals, allowing absence to be experienced as irreversible loss and no possibility of reunion in one episode and as geographical separation compatible with a possible future encounter in the other. The contrast thus illustrates how the same song may support different projection–appraisal processes, while leaving unresolved how comparable modulation would unfold with instrumental music alone or with songs whose lyrical content does not converge with the personal sense emerging within the episode.

These observations also suggest why Ren et al.’s (2024) findings on music and emotional memory can be extended theoretically. Ren and colleagues showed that affective music during recollection can influence the emergence of novel emotional memory traces aligned with the music’s valence. The present self-study concerns a different phenomenon: the immediate temporal organization of emotional recall while music is playing. It does not contradict experimental findings concerning valence congruence. Instead, it suggests that, under certain conditions, the emotional meaning of music depends less on the stimulus’s general valence and more on the situated personal sense projected onto it. The same music may therefore participate in different emotional directions depending on the object of recall, its personal sense, and appraisal processes.

## The Projection-Appraisal-Coupling Model

The self-study can be explained through a *projection-appraisal-coupling model*. The model has seven components: activity, object of thought, personal sense, projection onto music, appraisal, emotion, and feedforward into anticipation.

First, both episodes involved recall. It was not random mental wandering. It was a directed activity of remembering under musical conditions. Second, each activity had a distinct object of thought, its motive. Third, each object evoked a personal sense. Fourth, I projected each personal sense onto the song. Projection occurred because the song’s semantic openness allowed the thematic atmosphere of absence to be specified in different ways. Fifth, the projected personal sense was appraised. Sixth, emotion emerged from appraisal. Seventh, the emotion fed forward into the musical and cognitive processes, altering attention, intensifying recall, and shaping what could be anticipated next.

This model avoids two inadequate explanations. The first would say that the song itself caused the emotions. This cannot explain why the same song generated opposed states. The second would say that memories alone caused the emotions, with music merely accompanying them. This cannot explain, for example: (1) why in other circumstances I remembered my deceased father with pride, gratitude, and joy; (2) why in the specific recall activities the emotions unfolded with the song’s duration and phases; (3) why the musical atmosphere intensified the recall. A more adequate explanation is that emotion emerged through coupling. Music and the recall activity formed a dynamic system in which musical signals, personal sense, appraisal, and anticipation mutually shaped one another in a well-defined episode with beginning, middle, and ending phases, which I recognized as musical episodic modulation of emotion.

The word coupling is important because it indicates functional integration. Music was not outside the activity. It became part of the activity’s immediate conditions, functioning as a modulating agent. The song’s beginning helped establish the horizon of recall. Its development sustained and intensified appraisal. Its intensified middle phase coincided with the emotional peak (e.g., crying in the first episode). Its ending contributed to closure. For example, in the first episode, the end of the song coincided with the fading of sadness and the cessation of crying. This temporal correspondence alone is not sufficient to prove causality, but it supports the interpretation that music participated in the organization of the emotional episode.

The model also clarifies the role of familiarity. Because I knew the song well and valued it emotionally, I could anticipate its phases. Anticipation strengthened the coupling between music and recall. Familiar musical signals can become anticipators for cognitive and emotional processes. They prepare the listener for intensification, transition, or closure. In this sense, familiarity enables more precise temporal integration between music and activity. The model further explains why solitude mattered. Being alone provided conditions for emotional freedom. In the first episode, crying could unfold without social interruption. If another person had intervened by trying to comfort me, the episode might have been redirected.

Finally, the model emphasizes that appraisal may occur consciously or nonconsciously. In the self-study, I could identify explicit appraisals retrospectively, although not every aspect of the process was available for reflection in the moment. Musical modulation may operate through partial awareness: a listener may experience an affective change before being able to articulate the personal sense and appraisal that structured it. Future research should therefore combine first-person methods with behavioral measures, physiological indices, and experimental manipulation. Physiological measures could help identify candidate biomarkers of musical episodic modulation, although interpreting them would require convergent evidence linking physiological changes to the episode’s temporal organization. Relevant physiological measures include heart rate, respiration, electrodermal activity, and facial electromyography (Cacioppo et al., 2007; Grewe et al., 2007; Kreibig, 2010), complemented where appropriate by EEG (Cacioppo et al., 2007), fNIRS (Ferreri et al., 2015), or fMRI (Carlson et al., 2015). Given the complexity of the relationship between autonomic responses and emotion (Kreibig, 2010), physiological measures should be interpreted alongside experiential reports and behavioral observations within each episode.

**Fig. 1:**
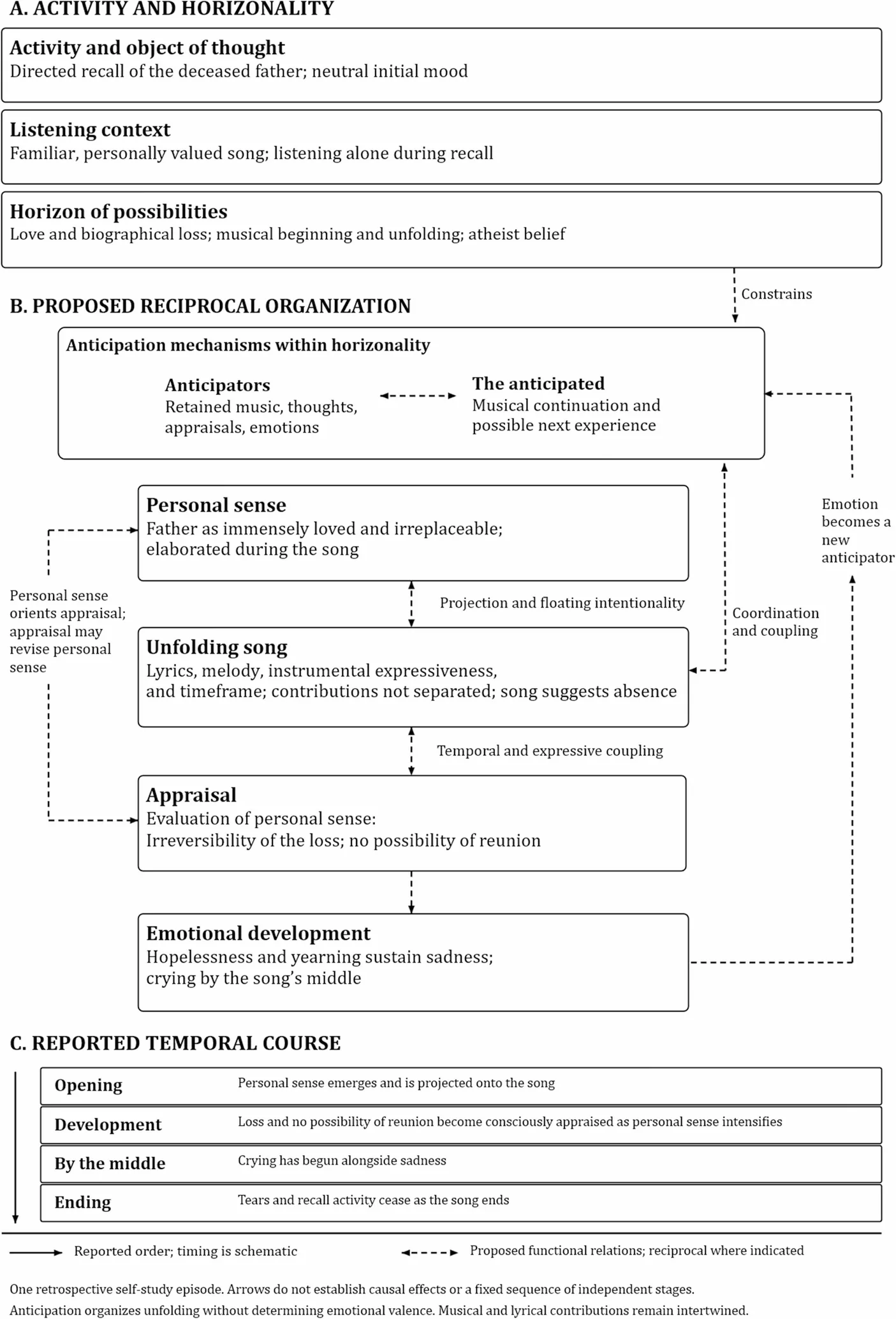
A model for musical episodic modulation of emotion in the first recall episode

Figure 1 presents a model for the temporal and reciprocal relationships among the major constructs in the first episode of musical modulation of emotion.

## Musical Phases and Action Organization

Although the self-study focused on recall, musical episodic modulation is not limited to emotional remembering. The broader theory proposes that music may also organize sequences of actions (Vieira, 2027). This possibility becomes clearer when a musical piece contains recognizable phases that can be coupled with corresponding phases of activity. A beginning may support preparation, a middle phase may support execution or development, and an ending may signal completion and evaluation. Evidence consistent with this broader organizing role comes from research showing that rhythmic auditory stimulation can influence motor planning and execution through auditory–motor entrainment (Repp & Su, 2013; Thaut et al., 2015).

Consider a hypothetical example inspired by ordinary domestic activity. A person assembles a family photo album while listening to a long instrumental piece with a slow and dense opening, a brighter, faster middle section, and a warm ending. The motive of the activity is to create a family album. The opening musical phase provides a nostalgic horizon that supports a preparatory action. The person remembers the father, appraises his absence, feels yearning, and decides to place his photos first, followed by the other family members in order of age. This decision is not purely cognitive. It is shaped by personal sense and emotion under the musical atmosphere.

When the music enters a brighter middle phase, the person begins the more operational action of selecting and arranging pictures. Because the criteria have already been established, faster musical energy supports procedural execution. While arranging photos, different family members become objects of thought, each evoking personal senses and appraisals: joy that the mother is alive, pride in siblings, regret about distance from a younger brother. The music’s middle phase supports movement through these operations and appraisals.

As the music enters its final phase, the person becomes oriented toward a completion action. The music ending functions as an anticipator of closure. The person notices that his own photographs have not yet been included and decides to place them at the end rather than reorganize everything. The final action is accompanied by an appraisal of life continuity, achievement, and gratitude. The album’s completion coincides with musical closure and emotional resolution.

This example illustrates how different musical phases may provide distinct temporal climates for activity. The term *climate* is used metaphorically to denote a temporary configuration of rhythmic, dynamic, and affective conditions that may make particular actions, thoughts, or appraisals more likely, accessible, salient, or affectively charged. These relationships are neither universal nor deterministic. Musical climates should therefore be understood as hypothesized and testable affordances, rather than as fixed correspondences between musical phases and actions. This cautious formulation is consistent with meta-analytic evidence showing heterogeneous effects of background music (Kämpfe et al., 2011).

The example also shows why music should not be reduced to background accompaniment. In a strongly coupled episode, the music contributes to decisions, pacing, emotions, and completion. It becomes part of the organization of activity. Musical signals and a person’s operations form changing immediate conditions for one another. What has just occurred in music prepares what may occur in action. And what has just occurred in action changes how the next musical signal is heard (Vieira, 2027). This bidirectional claim aligns with action-perception coupling work showing that prediction and sensorimotor integration shape both musical action and perception (Keller et al., 2014; Novembre & Keller, 2014).

The same principle can be applied to education, work, clinical practice, creative writing, film production, and everyday self-regulation (Vieira, 2027). However, applications must avoid simplistic prescriptions. The same music may modulate differently depending on activity demands and personal responsiveness. A person solving a difficult theoretical problem may require music that sustains a reflective horizon rather than music that accelerates action. A person coping with anxiety may benefit from music that stabilizes arousal, whereas another may experience the same music as emotionally intrusive.

## Discussion

The framework contributes to the psychology of music by shifting the question from isolated effects to temporal organization. The relevant issue is not only whether music changes mood, memory, attention, or conduct. It is how music may organize these dimensions together within lived episodes. This shift matters because the phenomenon concerns human functioning as culturally situated, temporally structured, and existentially charged.

Music is deeply cultural, carrying social histories and identity meanings (DeNora, 2000; Marett, 2005; Tagg, 1987; van Leeuwen, 1999). Yet music’s cultural meaningfulness does not eliminate personal variability. Rather, cultural meanings become available for personal sense-making. A romantic song, a funeral march, a hymn, a national anthem, a film theme, or a childhood song may operate differently across people and situations. The theory of musical episodic modulation accounts for this by placing personal sense and anticipation mechanisms at the center of the process.

The framework also contributes to phenomenological psychology by emphasizing the temporal structure of musical experience. Music modulates how feeling unfolds. In the first self-study episode, sadness did not appear as an instantaneous state. It was prepared by the song’s beginning, intensified through its development (e.g., crying), and ceased at its ending. In the second episode, hope and joy unfolded similarly over time. Such temporal organization is central to emotion as lived experience.

The theory further contributes to activity theory by showing how music can become part of an activity’s macrostructure and conditions. Leontiev’s activity-action-operation framework makes it possible to ask where music intervenes: it may or may not change an activity’s motive, but it can change how that motive is felt and personally sensed. It may sometimes help define, specify, or redirect the goal of an action, while also altering readiness, pace, salience, and affective orientation toward that goal. It may also support operations by cuing, sustaining, or temporally organizing their execution. Activity theory treats these units as dynamic and transformable rather than fixed compartments: an action can become an activity when it acquires an independent motive, and a routine operation can return to conscious action when conditions are disrupted (Leontiev, 1978).

In relation to memory research, the article suggests studying music-modulated recall not only through outcome measures, such as later memory accuracy or emotional traces, but also through microgenetic temporal analysis of recall episodes. Researchers might examine how musical phases correspond to shifts in the object of thought, imagery, and appraisal, as well as to changes in bodily response and narrative elaboration. Such work could combine phenomenological interviews (Petitmengin, 2006), physiological measures (Grewe et al., 2007), eye tracking during autobiographical retrieval (El Haj, 2024), neuroimaging of music-evoked autobiographical memory (Janata, 2009), and detailed analysis of musical structure (Egermann et al., 2013). This proposal builds on evidence that music can cue vivid autobiographical memories (Belfi et al., 2016), recruit autobiographical-memory networks (Janata, 2009), and influence the emotional content of later memory when affective music accompanies recollection (Ren et al., 2024).

Emotions arising during musical modulation are neither directly inscribed in musical structures nor stored in memories as fixed contents waiting to be released. Research distinguishing emotion perceived in music from emotion felt by listeners shows that musical expressiveness does not determine a single experienced emotion (Juslin & Västfjäll, 2008; Kallinen & Ravaja, 2006), while autobiographical-memory research conceptualizes remembering as a context-sensitive constructive process shaped by current goals and self-knowledge (Conway & Pleydell-Pearce, 2000). In the present account, emotion emerges as the listener appraises the personal sense of an object of thought under the temporal, affective, and situational conditions established by the ongoing musical episode. This position is compatible with appraisal theories, which hold that emotional differentiation depends on evaluations of relevance to concerns and goals, implications, agency, coping potential, and normative significance (Lazarus, 1991; Moors et al., 2013; Scherer et al., 2001). It is also compatible with psychological constructionist approaches, which treat emotional episodes as context-sensitive integrations of affective, interoceptive, conceptual, and prior experiential information (Barrett, 2017; Lindquist et al., 2012). Situated and scaffolded approaches further support treating music as a resource that participates in organizing an emotional episode, rather than as an external stimulus carrying a fixed emotional effect (Colombetti & Krueger, 2015; DeNora, 2000; Krueger, 2014). The present theory contributes to these traditions by specifying how musical temporality and coupling may organize the emergence and development of personal sense and the unfolding of appraisals within a bounded episode of recall.

### Conditions and Factors of Musical Episodic Modulation

The self-study and theoretical analysis distinguish three defining conditions of musical episodic modulation from additional factors that may facilitate or shape its occurrence. Within the proposed framework, the following three conditions are individually necessary and jointly sufficient to characterize the phenomenon; they are not presented as empirically established sufficient causal conditions. First, music must become functionally relevant to the episode and enter the activity horizon, whether through focal attention, peripheral engagement, or processes outside explicit awareness. Its floating intentionality enables context-sensitive integration, although its contribution varies with musical and lyrical constraints. When music has no functional relation to the unfolding activity, its mere presence does not constitute modulation. Second, music and activity must have some degree of temporal compatibility to be coordinated. Third, music must contribute to the episode’s temporal organization through anticipation-mediated coupling. Evidence aligned with these conditions includes music’s relevance and positive effects on work activities (Lesiuk, 2005), sensorimotor synchronization (Repp & Su, 2013), and musical prediction (Vuust et al., 2022).

The following factors may facilitate or shape modulation, but their specific forms are not universal requirements. First, familiarity and preference may strengthen modulation by facilitating anticipation and engagement, although unfamiliar music may also contribute when its features become functionally relevant to the episode. Second, the person’s history with music matters. Musical training, repeated listening, genre familiarity, and personal associations may influence anticipation and modulation. Third, the activity’s demands and structure matter. Routine motor actions and operations may be coupled more easily with rhythmic music, while reflective activities may require music that supports cognitive continuity without excessive interference. Fourth, social and environmental conditions matter. Solitude, collective coordination and synchronization, noise level, space, and interruption can alter the course of modulation. Fifth, the object of thought and personal sense are particularly relevant to the emotional recall pathway examined here. The same music may support different emotional trajectories depending on appraisals of what is remembered, desired, feared, or imagined. These proposed influences draw on evidence concerning task-dependent effects of preferred background music (Kiss & Linnell, 2021) and research on action–perception coupling that supports the roles of musical training and anticipation (Novembre & Keller, 2014). Work on joint musical action also highlights the importance of task structure for interpersonal coordination (Keller et al., 2014).

Sixth, willingness or openness to modulation may matter. People may resist some music, reject imposed sound, or experience it as invasive. Conversely, chosen music may be integrated more readily into self-regulation. Seventh, bodily state, mood, and affective needs shape the episode. Fatigue, anxiety, excitement, grief, pain, or calmness may affect how people appraise and use music. Eighth, sociocultural conditions matter. Musical signs are learned, shared, disputed, and transformed in social life. Taken together, these considerations are consistent with evidence that the everyday use of music depends on musical choice and sociocultural context (DeNora, 2000), that music-based mood regulation is shaped by listeners’ current affective needs and listening strategies (Saarikallio & Erkkilä, 2007), and that habitual music-listening strategies are associated with distinct behavioral and neural patterns of emotion regulation (Carlson et al., 2015).

These distinctions imply that musical episodic modulation should be studied as a relational phenomenon. It is not located only in music, only in the brain, only in the person, or only in culture. It emerges from the coupling of musical structure, embodied subjectivity, personal history, activity, and context. This relational view helps avoid both reductionism and relativism. Music has real structural features whose modulating effects depend on situated integration.

### Limitations

The present article has several limitations. The first is methodological. The self-study concerns one person and two episodes. It cannot establish general prevalence, causal mechanisms, or population-level patterns. Its value is exploratory and theoretical. It identifies a phenomenon, proposes concepts, and articulates mechanisms that future studies should test and refine. The second limitation concerns retrospective interpretation. Although the episodes were experientially vivid and analytically meaningful, the explanation was constructed through later reflection. Retrospective analysis may simplify, reorganize, or overinterpret lived experience, especially because verbal reports do not always accurately capture the processes that generated experience and action (Nisbett & Wilson, 1977). Future research should therefore use real-time or near-real-time experience sampling (Bolger & Laurenceau, 2013; Csikszentmihalyi & Larson, 1987), micro-phenomenological or phenomenologically oriented interviews (Høffding & Martiny, 2016; Petitmengin, 2006), physiological monitoring (Cacioppo et al., 2007), and behavioral observation or sequential analysis (Bakeman & Gottman, 1997) to examine how musical modulation unfolds.

The third limitation concerns the role of musical analysis. The present article refers to musical phases, tempo, affective atmosphere, familiarity, and semantic openness, but it does not provide a detailed musicological analysis of the song. Future work should examine how specific musical features, such as rhythm, harmonic progression, vocal timbre, melodic contour, dynamics, and formal structure, contribute to modulation. The fourth limitation concerns individual differences. I have a long history of using music for work, reflection, and self-regulation, and I am musically trained as a drummer. Other people may experience weaker, different, or even opposite forms of modulation. Some may find music distracting. Others may be strongly affected by lyrics but not by instrumental music. Individual differences must be central in future research.

The fifth limitation concerns the boundaries between music and language. The song used in the self-study includes lyrics, even though they were not quoted here. Lyrics may provide semantic cues that operate differently from purely instrumental music. Future studies should compare instrumental and lyrical music in recall activities. The sixth limitation concerns the theoretical integration of activity theory, phenomenology, appraisal theory, predictive coding, and dynamical systems. The framework is integrative, but more work is needed to specify how these theoretical traditions relate without collapsing one into another. Neural prediction does not replace personal sense; personal sense does not replace musical structure; musical structure does not replace cultural history. A pluralistic explanatory approach is required.

Finally, the proposed framework requires a distinction between conditions that may facilitate musical episodic modulation and observations that support its occurrence. Familiarity, preference, and thematic relevance may facilitate coupling, but none independently establishes that modulation has occurred. Likewise, emotional arousal, autobiographical recall, improved performance, or isolated temporal coincidences with musical events are insufficient evidence. Musical episodic modulation requires a temporally structured and functionally coherent relationship in which music contributes to the organization of an activity’s beginning, development, transitions, or closure. Cases in which music elicits a brief response without becoming integrated into the subsequent course of activity would therefore fall outside episodic modulation, although they may constitute other forms of musical influence. More challenging for the framework would be repeated observations that, under conditions independently identified as favorable to coupling, substantial changes in musical timing or phase structure leave the organization of thought, appraisal, emotion, and action unchanged, with that organization instead consistently only following task demands or internally generated transitions. Such findings would call for revising the proposed organizing role of musical temporality. Similarly, evidence that musical phases systematically organize an episode without any detectable anticipatory orientation across convergent experiential, behavioral, and physiological measures would require reconsideration of the central role assigned to anticipation mechanisms. An absence of consciously reported expectation alone would not establish such a challenge, since anticipation may operate outside explicit awareness. These distinctions prevent supportive conditions from being treated as evidence of the phenomenon and make the framework accountable to observations that may restrict its scope or require revision of its explanatory claims.

### Future Research Directions

Several lines of future research follow from the proposed framework. Complementary designs and methods could investigate the questions outlined below, including controlled experiments, studies of everyday activity, first-person inquiry, and computational modeling.

#### Musical Conditions, Emotional Recall, and Action Coordination

Studies could compare emotional recall under different musical conditions, including familiar, unfamiliar, preferred, non-preferred, lyrical, and instrumental music. Experiments could vary listening conditions to test their effects on reported emotional intensity, physiological responses, and subsequent emotional memory. Studies of everyday activity could examine how listeners coordinate their actions with musical duration and phases, using activity records and participants’ reports. Experimental changes to musical timing could then test whether the observed transitions in activity follow the altered musical organization.

#### Musical Expectations and Autobiographical Relevance

Predictive-processing accounts describe musical perception in terms of hierarchically organized expectations and their updating in response to prediction errors (Koelsch et al., 2019; Vuust et al., 2022). Future research could examine how musical expectations relate to personally significant possibilities within an activity. Anticipating a musical climax and anticipating a reunion are distinct processes; their proposed relationship requires empirical examination. Drawing on Cheung et al. (2023), computational models could estimate the predictability of musical events from their acoustic relationship to preceding sounds and their conformity to learned musical patterns. These estimates would provide a common reference across recall conditions involving the same music. Researchers could ask each listener to recall different personally significant people or events and examine whether the same musical moments are experienced differently. Reports of musical surprise and emotional experience, complemented by accounts of the recall episode, could help clarify how autobiographical relevance relates to musical expectancy.

#### Activity Macrostructure and Appraisal

The CODA model emphasizes how listeners’ goals and contextual concerns inform appraisal and the construction of emotional episodes during musical engagement (Lennie & Eerola, 2022). The proposed episodic framework extends this inquiry by examining how the macrostructure of human activity, comprising activity, action, and operation, relates to the unfolding of appraisal and emotion in coordination with music. Comparative studies could examine how the motive for remembering, the goal pursued during recall, and the conditions and means of remembering relate to the coordination between musical expectations and appraisal. Researchers could investigate naturally occurring variations or experimentally vary recall goals and listening conditions, examining whether and how these changes also affect participants’ motives for engaging in the activity. Participants’ accounts could help explain why remembering mattered to them, how they experienced and elaborated the personal sense of the recalled person or event during listening, and how they appraised that person or event as the episode unfolded. Behavioral records could document how they carried out the activity. A complementary experiment could bring forward or delay a musical transition, such as the onset of a climax, while keeping the recall instructions unchanged. Researchers could then examine whether reported changes in appraisal and emotional experience, alongside observable behavioral changes, also occur earlier or later relative to the altered transition. Comparing these patterns across motives, goals, and operational conditions could help clarify how the organization of the activity shapes temporal coupling. Together, these approaches would assess whether the macrostructure of human activity helps explain how music participates in the emergence, development, and transformation of appraisal and emotion within an episode.

#### Neural and Physiological Processes

Neurocognitive and psychophysiological studies could investigate candidate markers of musical episodic modulation, especially processes involving anticipation, reward, autobiographical memory, and episodic encoding and retrieval. These measures could be combined with experience records to examine temporal relationships, including under experimentally varied musical or activity conditions. Dopaminergic positron emission tomography combined with fMRI is particularly relevant because the anticipation of intense musical pleasure has been associated with greater caudate involvement, whereas peak pleasure has been associated with endogenous striatal dopamine release and greater nucleus accumbens involvement (Salimpoor et al., 2011; Zatorre & Salimpoor, 2013). In addition, pharmacological manipulation provided further evidence that dopamine contributes causally to musical pleasure and motivation (Ferreri et al., 2019). For the present theory, this distinction between anticipatory and peak responses suggests investigating whether reward-related activity varies with identifiable musical phases and corresponding transitions in the listener’s object of thought, appraisal, affect, or action. Nucleus accumbens responses to musical reward-prediction errors and its functional connectivity with auditory cortices could additionally be examined alongside behavioral measures to investigate how expected and unexpected musical events acquire motivational value and relate to learning within an episode (Gold et al., 2019; Salimpoor et al., 2013). Amygdala, hippocampal, and auditory-cortical responses to musical uncertainty and surprise may, in turn, help distinguish the processing of predictive deviations from more generalized affective arousal (Cheung et al., 2019).

Memory studies could examine how music participates in remembering and in forming new episodic memories. Measures of activity and connectivity within medial-temporal networks, including the hippocampus, and prefrontal and parietal networks could contribute to investigating music’s roles in retrieval, the encoding or binding of episodic elements, and the self-referential integration of autobiographical content (Janata, 2009; Rugg & Vilberg, 2013; Spaniol et al., 2009). Findings of altered lateral prefrontal activity during verbal encoding and retrieval in a musical context (Ferreri et al., 2015) also motivate studies combining fNIRS with memory performance measures and reports of remembering strategies to investigate how cognitive effort and associative strategies vary across musical phases.

Beyond memory, EEG and MEG could help identify the timing of neural responses to expected and unexpected musical events and investigate their relationships with changes in attention, emotional experience, and action (Vuust et al., 2022). Peripheral physiological measures, including heart rate and heart-rate variability, respiration, electrodermal activity, and facial electromyography, could track changes in bodily responses throughout the episode (Grewe et al., 2007). Neither autonomic responses nor activity in a single brain region would, by themselves, establish musical episodic modulation: autonomic responses are not uniquely specific to particular emotions, and individual brain regions may participate in several psychological processes (Kreibig, 2010; Poldrack, 2006). Neural and physiological findings should therefore be interpreted alongside behavioral measures and first-person reports, with attention to their temporal relationships with musical unfolding.

#### Phenomenological and Micro-Phenomenological Inquiry

Phenomenological and micro-phenomenological studies could investigate the lived temporal structure of music-modulated episodes and the horizons of anticipation experienced within them. Phenomenologically based interviews (Høffding & Martiny, 2016; Petitmengin, 2006) could explore how the object of thought emerged, how musical phases were experienced, when emotions intensified, how bodily sensations changed, and how closure occurred. Particular focus could be given to moments when an expected musical continuation became relevant to a developing thought, appraisal, or possible action. Interviews could also explore interruptions, unexpected transitions, and moments when music ceased to feel integrated with the activity. These inquiries could address naturally occurring episodes or experiences produced under controlled conditions. Their contribution would be to refine the projection-appraisal-coupling model through detailed descriptions, including experiences that suggest alternative interpretations.

#### Work, Learning, and Everyday Activity

Following Lesiuk’s (2005) investigation of music listening and work performance, future studies could examine how people use music to organize work, learning, and other everyday activities, including writing, studying, cleaning, exercise, and creation. Researchers could investigate whether listeners select music to initiate an activity, sustain engagement, coordinate routine operations, and how these uses vary with activity demands and musical familiarity. Activity records and participants’ reports could reveal relationships between musical phases and changes in pace, concentration, perceived effort, and decisions to continue or pause. These questions could be examined in everyday settings or through controlled comparisons of musical conditions within the same activity. Such studies could clarify when music supports the activity’s temporal organization and when it disrupts attention or conflicts with the listener’s goals.

#### Discourse and Collective Coordination

Discourse-oriented research (Adam, 2008; Bronckart, 1999; Gee, 1999; Melo & Vieira, 2025; Vieira & Kelly, 2014) could examine how music modulates verbal thinking and writing. Musical structures may relate differently to the pace, tone, and development of argumentative, explanatory, or narrative episodes. Analyses could connect musical transitions with pauses, topic shifts, and changes in discourse organization, combining writing records with participants’ accounts. This approach could also illuminate collective episodes in which people coordinate their talk and actions while jointly engaging with music. Analysis of turn-taking, prosody, gestures, and shared decisions could examine whether musical phases contribute to mutual anticipation and transitions between collective actions, while recognizing that participants may attribute different personal senses to the same music.

#### Film Production and Reception

Research on audiovisual aesthetics could explore productive and receptive musical modulation in film (Vieira, 2027). Film music may modulate spectators’ attention, perception, anticipation, and emotion, while music used during production may shape performance, gesture, camera movement, editing, and narrative pacing. Reception studies could compare responses to the same scene with different musical arrangements, examining perceived event boundaries, narrative expectations, and emotional interpretation. Production studies could investigate how performers and filmmakers coordinate their actions and decisions with music through observation, process records, and interviews. Distinguishing music used during filming from music introduced during editing would help specify its role in each phase. These approaches would extend the framework to cultural creation and reception while examining how musical and visual organization jointly contribute to an unfolding episode.

#### Adaptive Musical Systems

Computational and design-oriented research could develop adaptive musical systems that adjust tempo, intensity, repetition, or phase duration in relation to an unfolding activity. Evaluation could combine observations of use and participants’ feedback with controlled comparisons between adaptive music, fixed musical arrangements, and silence. These studies could examine whether the systems support activity organization and how users experience their interventions.

## Conclusion

This article proposed musical episodic modulation of human functioning as a theoretical framework for understanding how music may organize cognition, emotion, embodiment, and conduct within temporally bounded episodes. The central claim is that music becomes a modulating agent when its temporal, affective, and semantically open structure is functionally coupled with ongoing activity through anticipation mechanisms. The phenomenologically oriented self-study illustrated this process through two episodes of emotional recall involving the same song. In one episode, the song became coupled with the memory of a deceased father, supporting the appraisal of irreversible loss and no possibility of reunion, eliciting hopelessness, sadness, and yearning. In the other, it became coupled with the thought of a foreign woman, supporting appraisals of future romantic meeting and eliciting excitement, hope, and joy. The contrast shows that musical valence does not determine emotional outcome by itself. Emotion emerged through the situated projection and appraisal of personal sense within a musical horizon.

The broader theoretical contribution is to conceptualize music not only as an emotional stimulus, a background condition, or an object of perception, but also as a temporal organizer of human functioning. Musical modulation is not universal, automatic, or always beneficial. It depends on the dynamic relation between music, person, activity, culture, body, history, and context. Building on the broader theory of episodic modulation of human functioning and its elaboration in relation to music (Vieira, 2027), musical episodic modulation requires, as necessary and sufficient, that music acquire functional significance within an unfolding episode and contribute to its temporal organization through anticipation-mediated coupling. Together, these define the phenomenon within the proposed framework, rather than constituting empirically established sufficient causal conditions. Projection, personal sense elaboration, and appraisal specify the emotional recall pathway examined here, but are not mandatory components of every form of modulation. Music may also organize attention, bodily readiness, or action transitions without autobiographical projection or a distinct emotional change. Floating intentionality enables music to integrate meaningfully with ongoing activity because it does not necessarily refer to a single, predetermined object or situation. The listener’s concerns, objects of thought, and goals give the music personal significance, while its unfolding expressive structure shapes how the listener experiences those concerns, interprets those objects, and pursues those goals. Floating intentionality’s role therefore extends beyond emotional recall, although its contribution varies with musical and lyrical constraints. The framework thus proposes a common core of functional relevance, anticipation, and episodic coupling, with floating intentionality enabling context-sensitive pathways through which music participates in human functioning.

By integrating activity theory, phenomenology, floating intentionality, appraisal theory, and entrainment, the article provides a vocabulary for investigating music as part of the temporal architecture of lived experience. This framework opens a line of inquiry into how people use music to inhabit, regulate, intensify, transform, and complete episodes of human life.

## Data Availability

No datasets were generated or analysed during the current study.

## Declarations

### Note on Musical and Lyrical References

The article discusses the song only through concise analytical paraphrase and does not reproduce copyrighted lyrics.

## References

[CR1] Adam, J. M. (2008). *A linguística textual: Introdução à análise textual dos discursos [Text linguistics: Introduction to the text analysis of discourses]*. Cortez.

[CR2] Adeeb, E. R., & Vieira, R. D. (2024). Multi-level approach for critical discourse analysis: Boris Johnson’s statement on Ukraine to the House of Commons on 24 February 2022. *Revista de Estudos da Linguagem*, *32*(1), 66–86. 10.17851/2237-2083.32.1.66-86

[CR3] Bakeman, R., & Gottman, J. M. (1997). *Observing interaction: An introduction to sequential analysis* (2nd ed.). Cambridge University Press. 10.1017/CBO9780511527685

[CR4] Barrett, L. F. (2017). *How emotions are made: The secret life of the brain*. Houghton Mifflin Harcourt.

[CR5] Belfi, A. M., Karlan, B., & Tranel, D. (2016). Music evokes vivid autobiographical memories. *Memory (Hove, England)*, *24*(7), 979–989. 10.1080/09658211.2015.1061012 26259098

[CR6] Bogotá, J. D. (2025). Can the predictive mind represent time? A critical evaluation of predictive processing attempts to address Husserlian time-consciousness. *Phenomenology and the Cognitive Sciences*, *24*(4), 875–895. 10.1007/s11097-023-09898-y

[CR7] Bolger, N., & Laurenceau, J. P. (2013). *Intensive longitudinal methods: An introduction to diary and experience sampling research*. Guilford Press.

[CR8] Bronckart, J. P. (1999). *Atividade de linguagem, textos e discursos: Por um interacionismo sociodiscursivo [Activity of language, texts, and discourses: Toward a sociodiscursive interactionism]*. EDUC.

[CR9] Cacioppo, J. T., Tassinary, L. G., & Berntson, G. G. (Eds.). (2007). *Handbook of psychophysiology* (3rd ed.). Cambridge University Press. 10.1017/CBO9780511546396

[CR10] Carlson, E., Saarikallio, S., Toiviainen, P., Bogert, B., Kliuchko, M., & Brattico, E. (2015). Maladaptive and adaptive emotion regulation through music: A behavioral and neuroimaging study of males and females. *Frontiers in Human Neuroscience*, *9*, 466. 10.3389/fnhum.2015.00466 PMC4549560 26379529

[CR11] Céspedes-Guevara, J. (2023). A constructionist approach to emotional experiences with music. *Advances in Cognitive Psychology*, *19*(4), 46–62. 10.5709/acp-0402-4

[CR13] Cheung, V. K. M., Harrison, P. M. C., Meyer, L., Pearce, M. T., Haynes, J. D., & Koelsch, S. (2019). Uncertainty and surprise jointly predict musical pleasure and amygdala, hippocampus, and auditory cortex activity. *Current Biology*, *29*(23), 4084–4092e4. 10.1016/j.cub.2019.09.067 31708393

[CR12] Cheung, V. K. M., Harrison, P. M. C., Koelsch, S., Pearce, M. T., Friederici, A. D., & Meyer, L. (2023). Cognitive and sensory expectations independently shape musical expectancy and pleasure. *Philosophical Transactions of the Royal Society B: Biological Sciences*, *379*(1895). 10.1098/rstb.2022.0420 PMC10725761 38104601

[CR14] Clark, A. (2013). Whatever next? Predictive brains, situated agents, and the future of cognitive science. *Behavioral and Brain Sciences*, *36*(3), 181–204. 10.1017/S0140525X12000477 23663408

[CR15] Clarke, E. F. (2005). *Ways of listening: An ecological approach to the perception of musical meaning*. Oxford University Press. 10.1093/acprof:oso/9780195151947.001.0001

[CR16] Colombetti, G., & Krueger, J. (2015). Scaffoldings of the affective mind. *Philosophical Psychology*, *28*(8), 1157–1176. 10.1080/09515089.2014.976334

[CR17] Conway, M. A., & Pleydell-Pearce, C. W. (2000). The construction of autobiographical memories in the self-memory system. *Psychological Review*, *107*(2), 261–288. 10.1037/0033-295X.107.2.261 10789197

[CR18] Cross, I. (2005). Music and meaning, ambiguity and evolution. In D. Miell, R. MacDonald, & D. Hargreaves (Eds.), *Musical communication* (pp. 27–44). Oxford University Press. 10.1093/acprof:oso/9780198529361.003.0002

[CR19] Csikszentmihalyi, M., & Larson, R. (1987). Validity and reliability of the experience-sampling method. *The Journal of Nervous and Mental Disease*, *175*(9), 526–536. 10.1097/00005053-198709000-00004 3655778

[CR73] de Melo, V. F., & Vieira, R. D. (2025). Student judges’ criteria for evaluating classmate pre-service chemistry teachers’ arguments developed in a remote simulated jury activity. *International Journal of Science Education*, *47*(14), 1880–1922. 10.1080/09500693.2024.2376959

[CR20] De Picione, L., R (2015). The idiographic approach in psychological research: The challenge of overcoming old distinctions without risking to homogenize. *Integrative Psychological and Behavioral Science*, *49*(3), 360–370. 10.1007/s12124-015-9307-5 25939530

[CR23] de Warren, N. (2009). *Husserl and the promise of time: Subjectivity in transcendental phenomenology*. Cambridge University Press. 10.1017/CBO9780511657412

[CR21] DeNora, T. (2000). *Music in everyday life*. Cambridge University Press. 10.1017/CBO9780511489433

[CR22] Depraz, N., Varela, F. J., & Vermersch, P. (2003). On becoming aware: A pragmatics of experiencing. *John Benjamins*. 10.1075/aicr.43

[CR24] Eerola, T., & Vuoskoski, J. K. (2013). A review of music and emotion studies: Approaches, emotion models, and stimuli. *Music Perception*, *30*(3), 307–340. 10.1525/mp.2012.30.3.307

[CR25] Egermann, H., Pearce, M. T., Wiggins, G. A., & McAdams, S. (2013). Probabilistic models of expectation violation predict psychophysiological emotional responses to live concert music. *Cognitive Affective & Behavioral Neuroscience*, *13*(3), 533–553. 10.3758/s13415-013-0161-y 23605956

[CR26] El Haj, M. (2024). When you look at your past: Eye movement during autobiographical retrieval. *Consciousness and Cognition*, *118*., Article 103652. 10.1016/j.concog.2024.103652 38301389

[CR27] Engeström, Y. (1987). *Learning by expanding: An activity-theoretical approach to developmental research*. Orienta-Konsultit.

[CR28] Engeström, Y. (1999). Activity theory and individual and social transformation. In Y. Engeström, R. Miettinen, & R. L. Punamäki (Eds.), *Perspectives on activity theory* (pp. 19–38). Cambridge University Press. 10.1017/CBO9780511812774.003

[CR29] Engeström, Y. (2001). Expansive learning at work: Toward an activity theoretical reconceptualization. *Journal of Education and Work*, *14*(1), 133–156. 10.1080/13639080020028747

[CR30] Ferreri, L., Bigand, E., Bard, P., & Bugaiska, A. (2015). The influence of music on prefrontal cortex during episodic encoding and retrieval of verbal information: A multichannel fNIRS study. *Behavioural Neurology*, *2015*, Article707625. 10.1155/2015/707625 PMC4609813 26508813

[CR31] Ferreri, L., Mas-Herrero, E., Zatorre, R. J., Ripollés, P., Gomez-Andres, A., Alicart, H., Olivé, G., Marco-Pallarés, J., Antonijoan, R. M., Valle, M., Riba, J., & Rodriguez-Fornells, A. (2019). Dopamine modulates the reward experiences elicited by music. *Proceedings of the National Academy of Sciences*, *116*(9), 3793–3798. 10.1073/pnas.1811878116 PMC6397525 30670642

[CR32] Fiske, H. E. (1996). *Selected theories of music perception*. Edwin Mellen.

[CR33] Freud, S. (1962). Further remarks on the neuro-psychoses of defence. In J. Strachey (Ed.), *The standard edition of the complete psychological works of Sigmund Freud: Vol. 3. Early psycho-analytic publications* (pp. 157–185). Hogarth.

[CR34] Gebhardt, S., Kunkel, M., & von Georgi, R. (2014). Emotion modulation in psychiatric patients through music. *Music Perception*, *31*(5), 485–493. 10.1525/mp.2014.31.5.485

[CR35] Gee, J. P. (1999). *An introduction to discourse analysis: Theory and method*. Routledge. 10.4324/9780203019887

[CR36] Glassman, M. (1996). Understanding Vygotsky’s motive and goal: An exploration of the work of A. N. Leontiev. *Human Development*, *39*(6), 309–327. 10.1159/000278505

[CR37] Gold, B. P., Mas-Herrero, E., Zeighami, Y., Benovoy, M., Dagher, A., & Zatorre, R. J. (2019). Musical reward prediction errors engage the nucleus accumbens and motivate learning. *Proceedings of the National Academy of Sciences*, *116*(8), 3310–3315. 10.1073/pnas.1809855116 PMC6386687 30728301

[CR38] Grewe, O., Nagel, F., Kopiez, R., & Altenmüller, E. (2007). Emotions over time: Synchronicity and development of subjective, physiological, and facial affective reactions to music. *Emotion*, *7*(4), 774–788. 10.1037/1528-3542.7.4.774 18039047

[CR39] Gross, J. J. (2015). Emotion regulation: Current status and future prospects. *Psychological Inquiry*, *26*(1), 1–26. 10.1080/1047840X.2014.940781

[CR40] Grush, R. (2006). How to, and how not to, bridge computational cognitive neuroscience and Husserlian phenomenology of time consciousness. *Synthese*, *153*(3), 417–450. 10.1007/s11229-006-9100-6

[CR41] Gumperz, J. J. (1982). *Discourse strategies*. Cambridge University Press. 10.1017/CBO9780511611834

[CR42] Høffding, S., & Martiny, K. (2016). Framing a phenomenological interview: What, why and how. *Phenomenology and the Cognitive Sciences*, *15*(4), 539–564. 10.1007/s11097-015-9433-z

[CR43] Hommel, B. (2004). Event files: Feature binding in and across perception and action. *Trends in Cognitive Sciences*, *8*(11), 494–500. 10.1016/j.tics.2004.08.007 15491903

[CR44] Huang, R. H., & Shih, Y. N. (2011). Effects of background music on concentration of workers. *Work (Reading, Mass.)*, *38*(4), 383–387. 10.3233/WOR-2011-1141 21508527

[CR45] Huron, D. (2006). *Sweet anticipation: Music and the psychology of expectation*. MIT Press. 10.7551/mitpress/6575.001.0001

[CR46] Husserl, E. (1964). *The phenomenology of internal time-consciousness*. Indiana University Press.

[CR47] Janata, P. (2009). The neural architecture of music-evoked autobiographical memories. *Cerebral Cortex*, *19*(11), 2579–2594. 10.1093/cercor/bhp008 PMC2758676 19240137

[CR48] Juslin, P. N., & Västfjäll, D. (2008). Emotional responses to music: The need to consider underlying mechanisms. *Behavioral and Brain Sciences*, *31*(5), 559–575. 10.1017/S0140525X08005293 18826699

[CR49] Kallinen, K., & Ravaja, N. (2006). Emotion perceived and emotion felt: Same and different. *Musicae Scientiae*, *10*(2), 191–213. 10.1177/102986490601000203

[CR50] Kämpfe, J., Sedlmeier, P., & Renkewitz, F. (2011). The impact of background music on adult listeners: A meta-analysis. *Psychology of Music*, *39*(4), 424–448. 10.1177/0305735610376261

[CR51] Keller, P. E., Novembre, G., & Hove, M. J. (2014). Rhythm in joint action: Psychological and neurophysiological mechanisms for real-time interpersonal coordination. *Philosophical Transactions of the Royal Society B: Biological Sciences*, *369*(1658). 10.1098/rstb.2013.0394 PMC4240961 25385772

[CR52] Kiss, L., & Linnell, K. J. (2021). The effect of preferred background music on task-focus in sustained attention. *Psychological Research Psychologische Forschung*, *85*(6), 2313–2325. 10.1007/s00426-020-01400-6 PMC8357712 32748062

[CR53] Knight, W. E. J., & Rickard, N. S. (2001). Relaxing music prevents stress-induced increases in subjective anxiety, systolic blood pressure, and heart rate in healthy males and females. *Journal of Music Therapy*, *38*(4), 254–272. 10.1093/jmt/38.4.254 11796077

[CR54] Knutson, B., & Greer, S. M. (2008). Anticipatory affect: Neural correlates and consequences for choice. *Philosophical Transactions of the Royal Society B: Biological Sciences*, *363*(1511), 3771–3786. 10.1098/rstb.2008.0155 PMC2607363 18829428

[CR55] Koelsch, S. (2012). *Brain and music*. Wiley-Blackwell.

[CR56] Koelsch, S. (2014). Brain correlates of music-evoked emotions. *Nature Reviews Neuroscience*, *15*(3), 170–180. 10.1038/nrn3666 24552785

[CR57] Koelsch, S., Vuust, P., & Friston, K. (2019). Predictive processes and the peculiar case of music. *Trends in Cognitive Sciences*, *23*(1), 63–77. 10.1016/j.tics.2018.10.006 30471869

[CR58] Kreibig, S. D. (2010). Autonomic nervous system activity in emotion: A review. *Biological Psychology*, *84*(3), 394–421. 10.1016/j.biopsycho.2010.03.010 20371374

[CR59] Krueger, J. (2014). Affordances and the musically extended mind. *Frontiers in Psychology*, *4*, 1003. 10.3389/fpsyg.2013.01003 PMC3880934 24432008

[CR60] Large, E. W., & Jones, M. R. (1999). The dynamics of attending: How people track time-varying events. *Psychological Review*, *106*(1), 119–159. 10.1037/0033-295X.106.1.119

[CR61] Lazarus, R. S. (1991). Cognition and motivation in emotion. *American Psychologist*, *46*(4), 352–367. 10.1037/0003-066X.46.4.352 2048794

[CR62] Leman, M. (2007). *Embodied music cognition and mediation technology*. MIT Press. 10.7551/mitpress/7476.001.0001

[CR63] Lennie, T. M., & Eerola, T. (2022). The CODA model: A review and skeptical extension of the constructionist model of emotional episodes induced by music. *Frontiers in Psychology*, *13*, 822264. 10.3389/fpsyg.2022.822264 PMC9043863 35496245

[CR65] Leontiev, A. N. (1978). *Activity, consciousness, and personality*. Prentice-Hall.

[CR66] Leontiev, A. N. (1981). *Problems of the development of the mind*. Progress.

[CR64] Leontiev, A. A. (2006). Units and levels of activity. *Journal of Russian and East European Psychology*, *44*(3), 30–46. 10.2753/RPO10610405440303

[CR67] Lesiuk, T. (2005). The effect of music listening on work performance. *Psychology of Music*, *33*(2), 173–191. 10.1177/0305735605050650

[CR68] Lindquist, K. A., Wager, T. D., Kober, H., Bliss-Moreau, E., & Barrett, L. F. (2012). The brain basis of emotion: A meta-analytic review. *Behavioral and Brain Sciences*, *35*(3), 121–143. 10.1017/S0140525X11000446 PMC4329228 22617651

[CR69] London, J. (2004). *Hearing in time: Psychological aspects of musical meter*. Oxford University Press. 10.1093/acprof:oso/9780195160819.001.0001

[CR70] Luria, A. R. (1987). *The man with a shattered world: The history of a brain wound*. Harvard University Press.

[CR71] Marett, A. (2005). *Songs, dreamings, and ghosts: The wangga of North Australia*. Wesleyan University.

[CR72] Mayr, U., & Keele, S. W. (2000). Changing internal constraints on action: The role of backward inhibition. *Journal of Experimental Psychology: General*, *129*(1), 4–26. 10.1037/0096-3445.129.1.4 10756484

[CR74] Moors, A., Ellsworth, P. C., Scherer, K. R., & Frijda, N. H. (2013). Appraisal theories of emotion: State of the art and future development. *Emotion Review*, *5*(2), 119–124. 10.1177/1754073912468165

[CR75] Nardi, B. A. (Ed.). (1996). *Context and consciousness: Activity theory and human-computer interaction*. MIT Press. 10.7551/mitpress/2137.001.0001

[CR76] Nisbett, R. E., & Wilson, T. D. (1977). Telling more than we can know: Verbal reports on mental processes. *Psychological Review*, *84*(3), 231–259. 10.1037/0033-295X.84.3.231

[CR77] North, A. C., Hargreaves, D. J., & Hargreaves, J. J. (2004). Uses of music in everyday life. *Music Perception*, *22*(1), 41–77. 10.1525/mp.2004.22.1.41

[CR78] Novembre, G., & Keller, P. E. (2014). A conceptual review on action-perception coupling in the musicians’ brain: What is it good for? *Frontiers in Human Neuroscience*, *8*, 603. 10.3389/fnhum.2014.00603 PMC4139714 25191246

[CR79] Palmer, C., & Demos, A. P. (2022). Are we in time? How predictive coding and dynamical systems explain musical synchrony. *Current Directions in Psychological Science*, *31*(2), 147–153. 10.1177/09637214211053635 PMC8988459 35400858

[CR80] Patel, A. D. (2008). *Music, language, and the brain*. Oxford University Press. 10.1093/acprof:oso/9780195123753.001.0001

[CR81] Pelaprat, E., & Cole, M. (2011). Minding the gap: Imagination, creativity and human cognition. *Integrative Psychological and Behavioral Science*, *45*(4), 397–418. 10.1007/s12124-011-9176-5 21695391

[CR82] Pereira, C. S., Teixeira, J., Figueiredo, P., Xavier, J., Castro, S. L., & Brattico, E. (2011). Music and emotions in the brain: Familiarity matters. *Plos One*, *6*(11). 10.1371/journal.pone.0027241. Article e27241. PMC3217963 22110619

[CR83] Petitmengin, C. (2006). Describing one’s subjective experience in the second person: An interview method for the science of consciousness. *Phenomenology and the Cognitive Sciences*, *5*(3–4), 229–269. 10.1007/s11097-006-9022-2

[CR84] Poldrack, R. A. (2006). Can cognitive processes be inferred from neuroimaging data? *Trends in Cognitive Sciences*, *10*(2), 59–63. 10.1016/j.tics.2005.12.004 16406760

[CR85] Ren, Y., Mehdizadeh, S. K., Leslie, G., & Brown, T. (2024). Affective music during episodic memory recollection modulates subsequent false emotional memory traces: An fMRI study. *Cognitive Affective & Behavioral Neuroscience*, *24*(5), 912–930. 10.3758/s13415-024-01200-0 38955872

[CR86] Repp, B. H., & Su, Y. H. (2013). Sensorimotor synchronization: A review of recent research (2006–2012). *Psychonomic Bulletin & Review*, *20*(3), 403–452. 10.3758/s13423-012-0371-2 23397235

[CR87] Roman, I. R., Washburn, A., Large, E. W., Chafe, C., & Fujioka, T. (2019). Delayed feedback embedded in perception-action coordination cycles results in anticipation behavior during synchronized rhythmic action: A dynamical systems approach. *PLOS Computational Biology*, *15*(10). 10.1371/journal.pcbi.1007371. Article e1007371. PMC6822724 31671096

[CR88] Roth, W. M., & Lee, Y. J. (2007). Vygotsky’s neglected legacy: Cultural-historical activity theory. *Review of Educational Research*, *77*(2), 186–232. 10.3102/0034654306298273

[CR89] Rückriem, G. (2010). Digital technology, mediation, and activity theory. *Cultural-Historical Psychology*, *6*(4), 30–38. https://psyjournals.ru/en/journals/chp/archive/2010_n4/32813

[CR90] Rugg, M. D., & Vilberg, K. L. (2013). Brain networks underlying episodic memory retrieval. *Current Opinion in Neurobiology*, *23*(2), 255–260. 10.1016/j.conb.2012.11.005 PMC3594562 23206590

[CR91] Saarikallio, S., & Erkkilä, J. (2007). The role of music in adolescents’ mood regulation. *Psychology of Music*, *35*(1), 88–109. 10.1177/0305735607068889

[CR92] Salimpoor, V. N., Benovoy, M., Larcher, K., Dagher, A., & Zatorre, R. J. (2011). Anatomically distinct dopamine release during anticipation and experience of peak emotion to music. *Nature Neuroscience*, *14*(2), 257–262. 10.1038/nn.2726 21217764

[CR93] Salimpoor, V. N., van den Bosch, I., Kovacevic, N., McIntosh, A. R., Dagher, A., & Zatorre, R. J. (2013). Interactions between the nucleus accumbens and auditory cortices predict music reward value. *Science*, *340*(6129), 216–219. 10.1126/science.1231059 23580531

[CR94] Salvatore, S., & Venuleo, C. (2008). Understanding the role of emotion in sense-making: A semiotic psychoanalytic oriented perspective. *Integrative Psychological and Behavioral Science*, *42*(1), 32–46. 10.1007/s12124-007-9039-2 18293049

[CR95] Sannino, A., & Engeström, Y. (2018). Cultural-historical activity theory: Founding insights and new challenges. *Cultural-Historical Psychology*, *14*(3), 43–56. 10.17759/chp.2018140304

[CR96] Särkämö, T., Tervaniemi, M., Laitinen, S., Forsblom, A., Soinila, S., Mikkonen, M., Autti, T., Silvennoinen, H. M., Erkkilä, J., Laine, M., Peretz, I., & Hietanen, M. (2008). Music listening enhances cognitive recovery and mood after middle cerebral artery stroke. *Brain*, *131*(3), 866–876. 10.1093/brain/awn013 18287122

[CR97] Schäfer, T., Sedlmeier, P., Städtler, C., & Huron, D. (2013). The psychological functions of music listening. *Frontiers in Psychology*, *4*, 511. 10.3389/fpsyg.2013.00511 PMC3741536 23964257

[CR98] Scherer, K. R., Schorr, A., & Johnstone, T. (Eds.). (2001). *Appraisal processes in emotion: Theory, methods, research*. Oxford University Press. 10.1093/oso/9780195130072.001.0001

[CR99] See, C., Ng, M., & Ignacio, J. (2023). Effectiveness of music interventions in reducing pain and anxiety of patients in pediatric and adult emergency departments: A systematic review and meta-analysis. *International Emergency Nursing*, *66*, 101231. 10.1016/j.ienj.2022.101231 36528945

[CR100] Slevc, L. R., & Patel, A. D. (2011). Meaning in music and language: Three key differences. Comment on Towards a neural basis of processing musical semantics by Stefan Koelsch. *Physics of Life Reviews*, *8*(2), 110–111. 10.1016/j.plrev.2011.05.003 21570367

[CR101] Sloboda, J. A., & O’Neill, S. A. (2001). Emotions in everyday listening to music. In P. N. Juslin, & J. A. Sloboda (Eds.), *Music and emotion: Theory and research* (pp. 415–430). Oxford University Press. 10.1093/oso/9780192631886.003.0018

[CR102] Soto, D., Hodsoll, J., Rotshtein, P., & Humphreys, G. W. (2008). Automatic guidance of attention from working memory. *Trends in Cognitive Sciences*, *12*(9), 342–348. 10.1016/j.tics.2008.05.007 18693131

[CR103] Spaniol, J., Davidson, P. S. R., Kim, A. S. N., Han, H., Moscovitch, M., & Grady, C. L. (2009). Event-related fMRI studies of episodic encoding and retrieval: Meta-analyses using activation likelihood estimation. *Neuropsychologia*, *47*(8–9), 1765–1779. 10.1016/j.neuropsychologia.2009.02.028 19428409

[CR104] Stepp, N., & Turvey, M. T. (2010). On strong anticipation. *Cognitive Systems Research*, *11*(2), 148–164. 10.1016/j.cogsys.2009.03.003 PMC2827858 20191086

[CR105] Tagg, P. (1987). Musicology and the semiotics of popular music. *Semiotica*, *66*(1–3), 279–298.

[CR106] Terry, P. C., Karageorghis, C. I., Curran, M. L., Martin, O. V., & Parsons-Smith, R. L. (2020). Effects of music in exercise and sport: A meta-analytic review. *Psychological Bulletin*, *146*(2), 91–117. 10.1037/bul0000216 31804098

[CR107] Thaut, M. H., McIntosh, G. C., & Hoemberg, V. (2015). Neurobiological foundations of neurologic music therapy: Rhythmic entrainment and the motor system. *Frontiers in Psychology*, *5*, 1185. 10.3389/fpsyg.2014.01185 PMC4344110 25774137

[CR108] Valsiner, J. (2017). From methodology to methods in human psychology. *Springer*. 10.1007/978-3-319-61064-1

[CR109] van den Bosch, I., Salimpoor, V. N., & Zatorre, R. J. (2013). Familiarity mediates the relationship between emotional arousal and pleasure during music listening. *Frontiers in Human Neuroscience*, *7*, 534. 10.3389/fnhum.2013.00534 PMC3763198 24046738

[CR110] van Dijk, T. A. (1982). Episodes as units of discourse analysis. In D. Tannen (Ed.), *Analyzing discourse: Text and talk* (pp. 177–195). Georgetown University Press.

[CR111] van Leeuwen, T. (1999). *Speech, music, sound*. Macmillan. 10.1007/978-1-349-27700-1

[CR112] Varela, F. J., & Shear, J. (1999). First-person methodologies: What, why, how? *Journal of Consciousness Studies*, *6*(2–3), 1–14.

[CR113] Vieira, R. D. (2023). A modulação episódica pela música na cognição, emoção e comportamento humanos [Musical episodic modulation of human cognition, emotion, and behavior]. *Dialética*. 10.48021/978-65-252-9170-3

[CR114] Vieira, R. D. (2027). *Music and the episodic modulation of human functioning: A theoretical and exploratory study*. Springer.

[CR115] Vieira, R. D., & Kelly, G. J. (2014). Multi-level discourse analysis in a physics teaching methods course from the psychological perspective of activity theory. *International Journal of Science Education*, *36*(16), 2694–2718. 10.1080/09500693.2014.929754

[CR117] Vuust, P., & Witek, M. A. G. (2014). Rhythmic complexity and predictive coding: A novel approach to modeling rhythm and meter perception in music. *Frontiers in Psychology*, *5*, 1111. 10.3389/fpsyg.2014.01111 PMC4181238 25324813

[CR116] Vuust, P., Heggli, O. A., Friston, K. J., & Kringelbach, M. L. (2022). Music in the brain. *Nature Reviews Neuroscience*, *23*(5), 287–305. 10.1038/s41583-022-00578-5 35352057

[CR118] Weineck, K., Wen, O. X., & Henry, M. J. (2022). Neural synchronization is strongest to the spectral flux of slow music and depends on familiarity and beat salience. *eLife*, *11*, e75515. 10.7554/eLife.75515 PMC9467512 36094165

[CR119] Wertsch, J. V. (1981). The concept of activity in Soviet psychology: An introduction. In J. V. Wertsch (Ed.), *The concept of activity in Soviet psychology* (pp. 3–36). M. E. Sharpe.

[CR120] Zacks, J. M. (2020). Event perception and memory. *Annual Review of Psychology*, *71*(1), 165–191. 10.1146/annurev-psych-010419-051101 PMC8679009 31905113

[CR121] Zahavi, D. (2003). *Husserl’s phenomenology*. Stanford University Press. 10.1515/9781503620292

[CR122] Zatorre, R. J., & Salimpoor, V. N. (2013). From perception to pleasure: Music and its neural substrates. *Proceedings of the National Academy of Sciences*, *110*(Suppl. 2), 10430–10437. 10.1073/pnas.1301228110 PMC3690607 23754373

